# A Review of Botany, Phytochemistry, and Biological Activities of *Fragaria vesca* and *Fragaria viridis* Widespread in Kazakhstan

**DOI:** 10.3390/plants14132027

**Published:** 2025-07-02

**Authors:** Gayane A. Atazhanova, Gulnissa K. Kurmantayeva, Yana K. Levaya, Margarita Yu Ishmuratova, Marlen K. Smagulov

**Affiliations:** 1 Research Park of Biotechnology and Eco-Monitoring, Karaganda Buketov University, Universitetskaya Street, 28, Karaganda 100028, Kazakhstan; g-atazhanova@mail.ru (G.A.A.); kurmantaeva@qmu.kz (G.K.K.); margarita.ishmur@mail.ru (M.Y.I.); marlenkemel@mail.ru (M.K.S.); 2 School of Pharmacy, Karaganda Medical University, Gogol Street, 40, Karaganda 100017, Kazakhstan

**Keywords:** genus *Fragaria*, compounds, terpenoids, flavonoids, biological activity

## Abstract

According to current taxonomic consensus, the genus *Fragaria* L. (family Rosaceae) comprises nine recognized species: *Fragaria* × *ananassa* (Duchartre ex Weston) Duchesne ex Rozier, *Fragaria bucharica* Losinsk., *Fragaria viridis* subsp. *campestris* (Steven) Pael., *Fragaria chiloensis* (L.) Mill., *Fragaria moschata* Duchesne ex Weston, *Fragaria orientalis* Losinsk., *Fragaria vesca* L., *Fragaria virginiana* Mill., and *Fragaria viridis* Duchartre. Within the flora of Kazakhstan, two species are of particular significance: *F. vesca* L. and *F. viridis* Weston. The genus *Fragaria* L. is notable for its high content of diverse classes of biologically active compounds, which exhibit a broad spectrum of pharmacological and physiological activities. This review focuses on two *Fragaria* species native to the flora of Kazakhstan: *F. vesca* L. and *F. viridis* Weston. It summarizes recent advances in their botanical characterization, phytochemical profiling, extraction methodologies, and biological activities. Available evidence indicates that the phytochemical composition of extracts obtained from these species is modulated by a range of environmental and biological factors. These include habitat conditions, climate variability, chemotypic diversity, and the specific extraction protocols applied. Particular emphasis is placed on modern extraction techniques and the identification of low-molecular-weight metabolites. These include anthocyanins, volatile organic compounds, flavonoids, and phenolic acids, which contribute significantly to the observed biological effects. The review findings support the relevance of continued research into the potential application of these species as sources for the development of novel therapeutic and prophylactic agents. In addition, they highlight their promise for use in the formulation of biologically active compounds intended for food supplements and cosmetic products.

## 1. Introduction

Wild plant species that form part of the native flora have long served as important sources of medicinal and vitamin-rich raw materials, both for public use and for the pharmaceutical industry. Amid the growing global interest in natural and environmentally sustainable products, particular attention is being paid to their use in medicine and cosmetology. In this context, the identification and quality assessment of promising regional sources of medicinal plant materials has become increasingly relevant. The determination of phytochemical parameters in plant raw materials offers a scientific foundation for recommending specific species for cultivation and large-scale harvesting. This, in turn, enables the development of novel pharmaceutical and cosmetic products derived from these bioresources. The outcomes of phytochemical research on biologically active compounds may prove useful in both scientific and industrial fields. In addition, they can support the development of practical recommendations for local communities regarding the everyday use of wild and cultivated plant species.

Among the representatives of the genus *Fragaria* L. (family Rosaceae), particular research interest is directed toward two species: *F. vesca* L. and *F. viridis* Weston. Despite their morphological similarities, these species exhibit notable differences in the composition of phenolic compounds, organic acids, and other biologically active constituents [1]. The plants sources/their phytochemicals have been known already from ancient traditional medicine for their multiple effects, especially the bioactive compounds being used for their healing activity [2].

Representatives of the genus *Fragaria*, which are believed to have originated during the Tertiary period, are distributed across Eurasia and America. The diploid species, such as *F. vesca*, are considered among the most ancient representatives of the genus [3]. Eastern Asia is regarded as the primary center of origin and early diversification of *Fragaria*. It is in this region that both diploid and the earliest tetraploid species are thought to have emerged [4]. Subsequently, the genus *Fragaria* underwent extensive dispersal, expanding its range into Europe; America; and the mountainous regions of the tropics and Eurasia, including the Himalayas and Japan. In ancient Rus, *F. vesca* was harvested from the wild and used both as a food source and for medicinal purposes [5]. The natural range of *F. viridis* extends farther eastward, encompassing Central Asia and Siberia. Historically, it did not achieve the same wide distribution as *F. vesca*, although it was known to and used by humans at an earlier stage. Over time, both species began to be cultivated. This process led to the development of numerous hybrid cultivars [6].

Plants of the genus *Fragaria* are widely used in both conventional and traditional medicine as sources of medicinal and vitamin-rich raw materials [7]. Fresh berries, rich in vitamin C, are considered a valuable dietary remedy for liver, kidney, and heart diseases [8]. They are also prescribed in large quantities to weakened patients as a general tonic, as well as for the treatment of inflammatory conditions of the stomach and bile ducts, and to normalize intestinal function [9]. Infusions made from the fruits and leaves of *Fragaria* species are used as diuretics in the treatment of gout, urolithiasis, cholelithiasis, hypertension, scurvy, and other forms of vitamin deficiencies, as well as in cases of uterine bleeding [10]. Fresh berries, when consumed in large quantities, have demonstrated effectiveness in the treatment of vascular sclerosis, hypertension, constipation, and diarrhea. In the Republic of Belarus, a medicinal product known as “wild strawberry leaves” has been registered. In the Russian Federation, dietary supplements containing *Fragaria* are commercially produced. The quality of *F. vesca* raw material is regulated by the State Pharmacopoeia XIV edition (FS.2.5.0016.15) [11], which sets standards for the content of extractable substances (minimum 25%) and total flavonoids, expressed as rutin (minimum 1%).

The present review focuses on a comparative analysis of the chemical composition and biological activity of these two species growing in the territory of Kazakhstan. A review of the scientific literature shows that the chemical composition of *F. vesca* has been extensively studied. It is characterized by considerable diversity and variability. In contrast, the available data on the chemical composition of *F. viridis* leaves remain limited and fragmentary. This highlights a clear lack of in-depth and comprehensive studies on this species. Existing sources only briefly mention the presence of flavonoids, tannins, alkaloids, and vitamin C in the leaves of *F. viridis*, highlighting the need for further clarification and systematic investigation. The abundance of raw plant material, the presence of wild populations, the high biological productivity, and the potential for cultivation make these *Fragaria* species promising subjects for further scientific investigation. Moreover, *F. vesca* and *F. viridis* are among the most widespread *Fragaria* species in the flora of Kazakhstan. Their traditional use in phytotherapy further supports the rationale for selecting these species as objects of study. The aim of this review is to summarize and analyze current data on the chemical composition and biological activity of two *Fragaria* species that are of the greatest fundamental and applied significance. Particular emphasis is placed on their potential applications in the cosmetic, pharmaceutical, and food industries.

## 2. Methodology

For a comprehensive literature review, published data were analyzed through the following search engine systems: ScienceDirect^®^, PubMed^®^, SciFinder^®^, Web of Science^®^, Scopus^®^, and Google Scholar^®^. Additional information was obtained from academic dissertations, theses, specialized literature in plant sciences, national pharmacopoeias of various countries, and ethnobotanical sources. The data collection process adhered to the guidelines outlined in the Preferred Reporting Items for Systematic Reviews and Meta-Analyses statement [12]. During the search process, the following keywords were used: “*Fragaria*”, “compounds”, “isolation”, “extraction”, and “biological activity”. Previously published reviews provide data on the phytochemical and biological properties of *Fragaria* species up to 2019, indicating a lack of recent studies conducted over the past five years. In this regard, it is crucial to provide a more comprehensive overview and update the existing information on the chemical composition of *Fragaria* species. This includes botanical differences, biological properties of isolated extracts, essential oils, and individual compounds, as well as their potential applications in the pharmaceutical, cosmetic, and medical industries.

## 3. Botany

**Figure 1:**
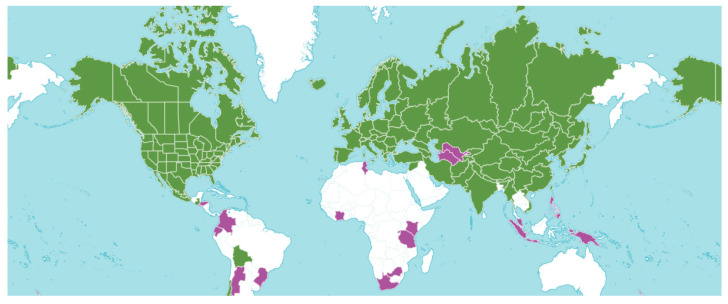
The wild (green) and cultivated (purple) distribution ranges of *Fragaria* L. species according to POWO [17].

The genus *Fragaria* L. belongs to the *Rosaceae* Juss. family and is represented by the cultivated species *F.* × *ananassa* (Duchartre ex Weston) Duchesne ex Rozier, as well as several wild species. Wild species are distributed across Eurasia and America, thriving in a variety of environments from the tundras of Canada and Alaska to the tropics of Eurasia. A small number of high-altitude species have been identified in the Himalayas, India, Japan, and the Kuril Islands. According to P.M. Zukovskij (1964) [13], the genus *Fragaria* L. is of typical forest and mesophytic origin. According to the Flora of the USSR [14], the genus includes nine species: *F.* × *ananassa* (Duchartre ex Weston) Duchesne ex Rozier, *F. bucharica* Losinsk., *F. viridis* subsp. *campestris* (Steven) Pael., *F. chiloensis* (L.) Mill., *F. moschata* Duchesne ex Weston, *F. orientalis* Losinsk., *F. vesca* L., *F. virginiana* Mill., and *F. viridis* Duchartre. For the flora of Kazakhstan [15], two species are reported: *F. vesca* L. and *F. viridis* Weston. M.S. Baitenov [16] also lists two species found in Kazakhstan, while the global flora includes approximately 15 species, distributed across Europe, Asia, and America. According to Plants of the World Online (POWO) [17,18], the genus *Fragaria* comprises 24 species, which are distributed across Eurasia and America. Introduced species are found in South America and Africa (Figure 1).

The genus was first described by Tragus in 1553, who referred to it as *fragaris*, derived from Latin, meaning “fragrant”. The systematic characterization was provided by Carl Linnaeus in 1753, while the taxonomy of the genus was further elaborated by Duschesne in 1766. According to various sources, the reported number of *Fragaria* species has ranged from 11 to 100. Currently, 24 species are officially recognized. It is believed that active hybridization processes are occurring between some species in the wild [13]. For instance, *F.* × *ananassa* is considered a cultivated hybrid, spontaneously produced through the crossbreeding of *F. chiloensis* (from Chile) and *F. virginiana* (from the Atlantic coast of America) [19]. This hybrid is the progenitor of all cultivated *Fragaria* cultivars. The center of origin of the genus is believed to be in Eastern and Southeastern Asia. From this region, *Fragaria* species subsequently spread to Europe and the Americas [20].

Members of the genus *Fragaria* are perennial plants that form rosettes of long-petioled leaves, which are typically compound and trifoliate. From these rosettes, creeping stolons emerge, which root at the nodes and facilitate effective vegetative propagation. These plants produce multi-flowered corymbs with either hermaphroditic or unisexual flowers. The genus includes both cultivated forms, which often revert to a wild state, and wild species that retain their natural characteristics.

An analysis of the morphological data for the two species, whose chemical composition and biological activity were the focus of this study, revealed several differences. The primary differences involve plant size, the shape of leaflets and teeth, the characteristics of inflorescences and flowers, and the presence of hermaphroditic or unisexual flowers, as well as the shape and coloration of petals, sepals, and fruits. All the studied species primarily inhabit forest, meadow, or shrub communities of the mesophytic type.

*F. vesca* is characterized by light green, thin leaves with more pronounced teeth. The flower stems are long and often extend significantly above the leaves. The berries are elongated, red, with a soft texture and a distinct, strong fragrance. The tendrils of *F. vesca* are well-developed and play an active role in vegetative reproduction, forming new rosettes. In its natural habitats in Kazakhstan, this species is found on the edges and clearings of island forests, in spruce and fir forests, and in shrub thickets. Its distribution range spans the Akmolinsk, Almaty, East Kazakhstan, North Kazakhstan, and Karaganda regions, as well as the areas surrounding Tarbagatai, the Dzhungarian Alatau, the Chu-Ili Mountains, the Kyrgyz Alatau, Western Tien Shan, and other regions [15].

**Figure 2:**
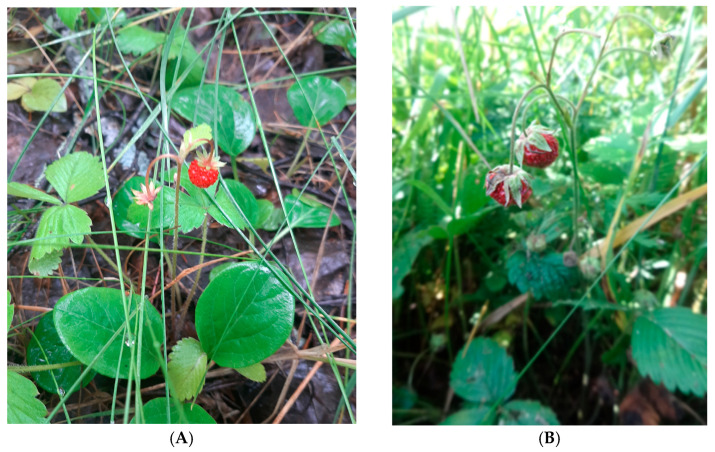
(**A**) Photograph of *Fragaria vesca* growing in Central Kazakhstan. (**B**) Photograph of *Fragaria viridis* growing in Central Kazakhstan. Original photos archived by Ishmuratova M. Yu.

**Figure 3:**
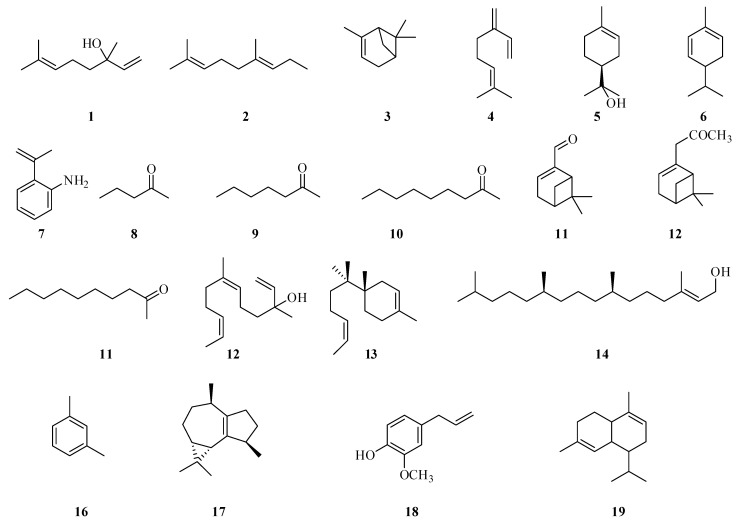
Volatile oils from *Fragaria*.

The flowering period of *F. vesca* and *F. viridis* in Kazakhstan occurs from May to June, while fruiting is observed from June to July (Figure 2 and Figure 3).

In *F. viridis*, the leaves are dark green, dense, with less pronounced teeth. The flower stems are short, and the flowers are often located below the level of the leaves. The berries are more rounded and sometimes remain greenish even when fully ripe. They have a firmer texture and a less pronounced aroma. The tendrils are fewer, and reproduction is predominantly through seeds. *F. viridis* grows in aspen-birch thickets; on open grassy mountain slopes, forest edges, and clearings; and in meadows and meadow steppes. Its distribution range is smaller than that of *F. vesca*, but the local population also uses its fruits for consumption in fresh, cooked, and dried forms.

## 4. Methods for Obtaining Extracts and Essential Oils from *Fragaria* Species

Plants of the genus *Fragaria* (*Fragaria* spp.) represent a valuable source of low-molecular-weight bioactive compounds. These include phenolic constituents, anthocyanins, flavonoids, organic acids, vitamins, and various other phytochemicals. Such metabolites are known to exhibit significant antioxidant, anti-inflammatory, and antimicrobial activities. The selection of an optimal extraction method is critical for the preservation of these compounds. Moreover, it plays a pivotal role in ensuring their effective application across pharmaceutical, food, and cosmetic industries.

Extraction methods for isolating bioactive compounds from *Fragaria* species can be classified according to several criteria. These include the type of solvent employed, the mode of physical treatment applied to the plant material, and the overall process technology. Conventional techniques encompass maceration, percolation, and Soxhlet extraction. In contrast, modern approaches involve ultrasound-assisted extraction, microwave-assisted extraction, and supercritical fluid extraction. In addition, biotechnological strategies are increasingly utilized. These include enzymatic hydrolysis and the use of cell suspension cultures.

Maceration involves soaking plant material in an appropriate solvent at room temperature or under mild heating. The efficiency of this method is influenced by several parameters, including extraction duration, temperature, and the ratio of plant biomass to solvent volume. Among the key advantages of maceration are its simplicity and low operational costs. For instance, fruits of *Fragaria* spp. have been extracted using 96% ethanol over a period of 48 h. The resulting extract was subsequently filtered and concentrated using a rotary evaporator at 37 °C until a viscous mass was obtained [21]. However, the principal limitation of maceration lies in the extended processing time required to achieve adequate yields.

In some studies, the maceration process has been conducted over extended durations. For example, extraction at 25 °C without agitation was carried out for a period of two weeks. The solid-to-solvent mass ratio was maintained at 1:10. Upon completion, the solution was filtered using Macherey–Nagel filter paper. The solvent was then evaporated, and the remaining biomass was resuspended in dimethyl sulfoxide to a final concentration of 100 g/L [22].

Modified short-duration protocols have also been developed to enhance process efficiency. In one such approach, dried leaves of *F. vesca* were extracted using a 50% aqueous ethanol solution at a solid-to-liquid ratio of 1:10 (*w*/*v*). The extraction was performed at room temperature under magnetic stirring at 750 rpm for one hour. Following vacuum filtration, ethanol was removed using a rotary evaporator. The remaining aqueous phase was subsequently lyophilized and stored at 22 °C [23].

Since the leaves of *F. vesca* contain various classes of phenolic compounds, research efforts continue to focus on developing more efficient extraction methods. Additionally, improving analytical techniques for characterizing these bioactive components remains a priority. To evaluate the effects of temperature, extraction time, and solvent-to-sample ratio, both microwave-assisted and pressurized solvent extraction methods have been tested. The highest total phenolic content and strongest radical-scavenging activity were achieved using ultrasound-assisted extraction. This was performed at 150 °C for 5 min, with a solvent-to-biomass ratio of 40:1 (*v*/*w*). Microwave-assisted extraction at 80 °C for 5 min was particularly effective for isolating phenolic acids. In contrast, ultrasound-assisted extraction resulted in a higher yield of proanthocyanidins [24].

Other studies investigated the impact of ultrasound extraction duration and solvent composition on the efficiency of proanthocyanidin recovery from *Fragaria* leaves. The optimal conditions were identified as follows: extraction with a 56% acetone–water mixture, combined with ultrasonic treatment for 50 min in a bath operating at a frequency of 35 kHz. Under these parameters, the maximum yield of proanthocyanidins reached 124.0 mg per 100 g of dry weight [25].

Among the modern high-efficiency extraction techniques, the Dionex™ ASE 350 (Thermo Scientific™, Salt Lake City, UT, USA) accelerated solvent extraction system stands out as a powerful alternative to Soxhlet, ultrasound-assisted, and supercritical fluid extraction methods. This technique relies on the application of elevated pressure and temperature within sealed stainless-steel cells. Such conditions significantly accelerate the recovery of target compounds. For example, extraction of *F.* × *ananassa* was carried out using ethanol and water at temperatures of 90 °C and 110 °C, respectively, under a pressure of 10.3 MPa. Conventional maceration was performed in parallel for comparison. The results demonstrated that accelerated solvent extraction reduced the extraction time from several hours to just 5–20 min. It also doubled the extract yield, minimized solvent consumption, and ensured high reproducibility of the results [26].

In addition to extracts, various types of oils are also obtained from *Fragaria* plants. The extraction method plays a critical role in determining both the composition and quality of these oils. Cold pressing preserves the nutritional and functional properties of the oil. However, it is associated with a relatively low yield up to 15% of the oil remains in the press cake and high susceptibility of the product to oxidation. An alternative approach involves supercritical carbon dioxide extraction. This method is carried out under anaerobic conditions and enables near-complete recovery of lipid components. A comparative study was conducted on *F. vesca* seed oil obtained via both methods, followed by a detailed compositional and quality assessment [27].

To prepare aqueous extracts from *F. vesca* leaves, the authors employed two approaches: decoction and infusion. In the decoction method, 1 g of dried plant material was mixed with 50 mL of ultrapure water, boiled for 15 min, then steeped for an additional 20 min before filtration. In the infusion method, the plant material was poured over with water preheated to 95 °C and steeped for 20 min. Both aqueous extracts exhibited notable biological activity and hold promise for use in the development of functional foods and cosmetic formulations [28].

Research showed that extraction conditions significantly influence the structural characteristics of the resulting polyphenol–polysaccharide conjugates. When high temperatures, ultrasound, or microwave irradiation are applied, significant changes occur in the structure of the isolated polysaccharides. These changes happen despite the medium’s consistent acidity. These methods provide higher extraction efficiency and enhanced anticoagulant activity compared to traditional techniques. Additionally, they enable the isolation of compounds with lower molecular weight [29].

The authors proposed the use of membrane ultrafiltration to obtain high-purity dietary fibers composed of polyphenol–polysaccharide conjugates from *F. vesca*. In one protocol, 250 g of ground plant material was steeped in a 0.1 M NaOH solution for 24 h, followed by extraction at 97 °C for 6 h. The resulting solution was then neutralized, concentrated, and subjected to ultrafiltration. This approach significantly reduced extraction time and the consumption of organic solvents and water, while maintaining high efficiency in recovering the target compounds [30].

Volatile compounds are typically isolated using methods based on the analysis of the vapor phase, including solid-phase microextraction, steam distillation, supercritical fluid extraction, and dynamic headspace techniques [31]. Solid-phase microextraction is the most widely used method for extracting aromatic compounds. It is especially preferred for analyzing fresh fruits without causing their destruction. It is often coupled with gas chromatography and allows for qualitative assessment of volatile substances. Steam distillation, however, is less sensitive to thermolabile compounds, while supercritical fluid extraction offers a gentle approach for extracting terpenes and esters. Dynamic headspace technique is used to capture volatile substances as they transition into the gas phase. It is important to note that methods for analyzing volatile compounds do not provide a complete profile of the plant matrix. Rather, they offer an assessment of the presence of specific volatile components.

## 5. Volatile Components of *Fragaria vesca* and *Fragaria viridis*

Over 20 wild species of *Fragaria* have been identified, with the most common being the diploid species *F. vesca* and *F. viridis*. Studies focusing on the volatile aromatic compounds of *Fragaria* have revealed that the fruits contain a variety of low-molecular-weight substances responsible for their characteristic aroma. Among these, complex esters, aldehydes, ketones, alcohols, and terpene compounds stand out. The composition and concentration of these compounds can vary significantly depending on the cultivar and the ripeness of the fruit. The presence of specific compounds is influenced by factors such as berry maturity, cultivation conditions, and the storage period and conditions. Based on differences in the composition of aroma-forming compounds, researchers have made numerous attempts to identify characteristic markers. These markers are intended to distinguish not only between *Fragaria* species but also among individual cultivars. The most commonly encountered volatile components in the two *Fragaria* species are presented in Table 1.

Volatile compounds in *Fragaria* are key determinants of its characteristic aroma and flavor profile. For instance, the monoterpenoids linalool (**1**) and geraniol (**2**) contribute to the freshness and complexity of the aroma. Lactones impart tropical nuances such as peach and coconut notes, while aldehydes are responsible for fresh and herbaceous undertones. A distinctive feature of the volatile profile of *F. vesca* fruit is its richness in esters and monoterpenes. These include α-pinene (**3**), β-myrcene (**4**), α-terpineol (**5**), and α-phellandrene (**6**). Together, they contribute to the pleasant and readily recognizable aroma of *F. vesca*, which is closely associated with methyl 2-aminobenzoate (**7**).

Berry aroma is influenced by both genotype and environmental conditions [32,33,34,35,36,37,38,39,40,41,42]. Volatile organic compounds are primarily responsible for aroma and also contribute significantly to the flavor of fresh berries. Although these compounds represent only 0.01% to 0.001% of the fresh fruit weight, they have a profound impact on fruit quality. Approximately 360 volatile compounds have been identified from fresh *Fragaria* spp. material. These include esters, aldehydes, ketones, alcohols, terpenes, furanones, and sulfur-containing compounds. Among them, esters are the most abundant and diverse class, both qualitatively and quantitatively. A total of 131 different esters have been identified in the aroma profile of *Fragaria*. Esters impart fruity and floral notes and account for 25% to 90% of the total volatile content in fresh, ripe fruits. Other compound classes such as aldehydes and furanones may comprise up to 50% of the volatile fraction. Alcohols can make up to 35%, though they typically contribute less to aroma perception. Terpenes usually constitute less than 10%, and sulfur compounds less than 2% of volatiles. Despite their low abundance, both classes can significantly influence the overall aroma of *Fragaria*.

In contrast to cultivated garden varieties, *F. vesca* is renowned for its intense flavor and aroma. Most wild *Fragaria* species produce relatively small fruits, yet these accumulate higher levels and a broader spectrum of volatile molecules compared to domesticated cultivars [42]. Due to its rich aromatic profile, *F. vesca* has attracted significant scientific interest. Methyl anthranilate (**7**) is recognized as the primary aroma compound in *F. vesca*. Ketones such as 2-pentanone (**8**), 2-heptanone (**9**), and 2-nonanone (**10**), as well as terpenoids including myrtenal (**11**), myrtenyl acetate (**12**), and α-terpineol (**5**), are found at higher concentrations in wild berries [43].

The high variability of volatile compounds in *Fragaria* can be attributed to genetic factors, ripening stage, and post-harvest conditions. Compared to cultivated strawberries, *F. vesca* exhibits a distinctive and more intense flavor profile. Genetic factors have also been shown to influence its aroma characteristics. In a study by Urrutia [44], the genetic basis of aromatic compounds in *F. vesca* fruit was explored. This species was found to possess a richer and more fruit-forward aroma than that of cultivated varieties. The study utilized a collection of nearly isogenic lines derived from a cross between *F. vesca* and *F. bucharica*. This genetic material enabled detailed investigation of variability and qualitative traits in fruit composition. Volatile compounds were analyzed using gas chromatography–mass spectrometry (GC-MS), which revealed a complex and highly variable volatile profile. A total of 100 compounds were identified, including esters, aldehydes, ketones, alcohols, terpenoids, furans, and lactones. Within this dataset, a subset of key volatiles was identified as major contributors to the characteristic aroma and flavor of *F. vesca*. Genetic analysis revealed 50 major quantitative trait loci.

To assess aroma quality based on chemical composition, volatile compounds are typically grouped according to their olfactory impact on berry aroma. Among these, esters form one of the principal classes of aroma-active compounds, imparting the characteristic fruity scent to the fruit. According to studies [32,33,34,35,36,37,38,39,40,41,42], esters account for approximately 25% to 90% of the total volatile content in *Fragaria*. These studies also indicate that nearly all esters identified in *Fragaria* are saturated. This means that neither the alcohol nor the acid fragments of these molecules contain double bonds. Differences in the composition of volatile esters between wild and cultivated *Fragaria* species have been reported by Donga et al. [37]. The authors proposed that the ester profile, dominated by acetate esters in *F. Vesca* and by ethyl hexanoate (**13**) in *F.* × *ananassa*, plays a key role in defining the divergent aromatic patterns of the two species.

Aroma-forming compounds characteristic of *Fragaria* cultivated in regions such as the United States and China are also present in the volatile profile of *Fragaria* grown in the Republic of Belarus. These include esters, terpenes, furans, and lactones. In *Fragaria* cultivated in this region, the volatile fraction includes not only saturated esters but also unsaturated ones. These unsaturated esters contain double bonds in both the alcohol and acid moieties of the ester molecule. Volatile compound analysis revealed a striking relationship between berry pigmentation and ester composition. Fruits harvested from the same plant at the same time but differing in color intensity displayed distinct volatile profiles. Light red berries showed a higher proportion of unsaturated esters, whereas dark red berries were richer in saturated esters. For example, floral-scented compounds such as β-linalool (**1**) and nerolidol (**12**) were found in significantly greater concentrations in dark red berries compared to light red ones [31].

Sensory evaluation of *F. viridis* fruits revealed a pleasant taste and an exceptionally fresh, fruity aroma. Essential oils from the leaves and fruits of *F. viridis* were obtained via hydrodistillation and subsequently analyzed by GC-MS. In the leaf essential oil, 39 constituents were identified, accounting for 67.3% to 80.7% of the total oil composition. The major compounds included β-linalool (**1**) at levels up to 8.9%; n-nonanal (**14**) (0.5–8.6%); nerolidol (**12**) (2.1–4.8%); α-bisabolol (**15**) (0.8–6.7%); and phytol (**16**), whose concentration ranged from 18.4% to 47.4% (Figure 3). Notably, the relative abundance of these components varied depending on the growing conditions. The essential oil extracted from *F. viridis* fruits contained 34 compounds, which together comprised 42.0% to 70.7% of the total oil content. The dominant constituents included m/p-xylene (**17**) (2.4–14.0%), isolidene (**18**) (4.7–8.5%), methyl eugenol (**19**) (3.3–8.4%), and α-muurolene (**20**) (6.8–11.3%). Additionally, one unidentified compound was detected, with a concentration ranging from 0% to 25.6%, depending on the environmental conditions [33].

Thus, the aroma of *Fragaria* results from a complex interplay of the concentrations and ratios of various volatile compounds, which are directly influenced by both genetic factors and cultivation conditions.

## 6. Phenolic Profile of *Fragaria*

The value of *Fragaria* berries is largely attributed to their high content of phytochemical compounds, primarily phenolic substances. Among the richest dietary sources of phenolics, *Fragaria* ranks ninth, providing approximately 390 mg of total polyphenols per serving. The sustained global increase in *Fragaria* consumption underscores the promising prospects for its future production and commercialization [4]. Among the low-molecular-weight metabolites present in *Fragaria*, phenolic compounds represent the most abundant and diverse class. Flavonoids constitute the principal subgroup of phenolics, with anthocyanins being predominant. Other flavonoid subclasses such as flavanols and flavonols contribute to a lesser extent. In addition to flavonoids, *Fragaria* phenolics also include hydrolyzable tannins, primarily ellagitannins and gallotannins, as well as phenolic acids, including hydroxybenzoic and hydroxycinnamic acids.

### 6.1. Anthocyanins in Fruits of Fragaria vesca and Fragaria viridis

**Figure 4:**
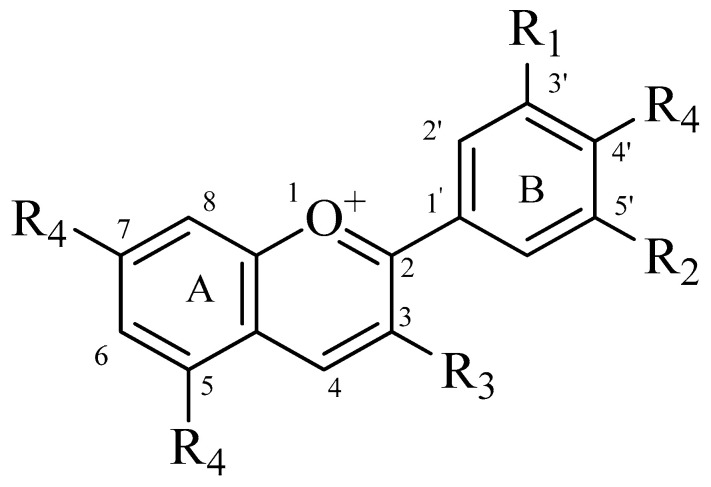
Flavylium cation structure of anthocyanins. R_1_ and R_2_ represent H, OH, or OCH_3_; R_3_ denotes a glycosyl group or hydrogen; R_4_ is either OH or a glycosyl substituent.

Anthocyanins, the water-soluble glycosides of anthocyanidins, are widely distributed polyphenolic secondary metabolites belonging to the flavonoid class. These compounds are derivatives of 2-phenylbenzopyran (or 2-phenylchroman) and structurally represent glycosides of flavylium cations-anthocyanidins (Figure 4). The core structure of anthocyanidins consists of a C6–C3–C6 carbon skeleton. It comprises a chromane ring system, substituted at position 2 with a phenyl ring. In this structural framework, the substituted benzene ring directly fused to the chromane is designated as ring A. The phenyl ring attached at position 2 is referred to as ring B, and the heterocyclic chromane ring itself is identified as ring C. A key structural feature distinguishes anthocyanins from other flavonoid subclasses. It is the presence of a formal positive charge on the oxygen atom located within the pyran ring.

Anthocyanins exhibit a broad spectrum of pharmacological activities, as demonstrated in rigorously controlled in vitro and in vivo experiments, as well as in preclinical and clinical studies. These compounds have been shown to enhance ocular trophism and night vision, reduce the risk of cardiovascular diseases, and mitigate vascular complications associated with type 2 diabetes mellitus. Such effects are primarily attributed to their antioxidant capacity, as well as their hypoglycemic and hypolipidemic properties. Additionally, anthocyanin-rich extracts have been reported to exert anti-inflammatory effects, inhibit lipid peroxidation, decrease capillary permeability and fragility, and stabilize cellular membranes.

Anthocyanins are the most well-known and quantitatively significant class of polyphenolic compounds in *Fragaria*. They are of considerable biomedical interest due to a broad range of health-promoting properties, including antioxidant potential, anticancer activity, anti-inflammatory effects, and antiangiogenic mechanisms of action [45]. The quantitative composition of the anthocyanin profile is primarily determined by the *Fragaria* genotype. According to the Joint World Health Organization Expert Committee on Food Additives, the acceptable daily intake of anthocyanins for humans has been set at 2.5 mg per kg of body weight. In contrast, Russian nutritional guidelines recommend a daily intake of 50 to 150 mg of anthocyanins to support health and physiological function [46].

More than 25 distinct anthocyanin pigments have been identified in *Fragaria* spp., yet pelargonidin-3-glucoside (**21**) consistently emerges as the predominant anthocyanin, regardless of genetic background or environmental conditions [47]. Cyanidin-3-glucoside (**22**) is also a constant constituent of *Fragaria*, although it is typically present in lower concentrations. In addition to these principal compounds, several minor anthocyanins have been detected in strawberry fruits, including pelargonidin-3-rutinoside (**23**) and pelargonidin-3-arabinoside (**24**). When comparing the average anthocyanin content across different samples, a significantly higher concentration was observed in wild-collected *F. vesca* fruits compared to cultivated varieties, 132 mg per 100 g^1^ versus 90 mg per 100 g^1^, respectively [48].

Anthocyanin accumulation is also influenced by environmental factors, including abiotic stressors and exogenous compounds. This suggests that targeted manipulation of environmental conditions could enhance anthocyanin content in strawberry fruits. Plants grown under field conditions are frequently subjected to multiple stress factors throughout their development. These effects are further exacerbated by abrupt shifts in global climate patterns. For instance, findings reported in [49] demonstrate that water deficit positively affects anthocyanin accumulation in *F. vesca* berries, but only when ambient temperatures remain moderate, around 20 °C.

**Figure 5:**
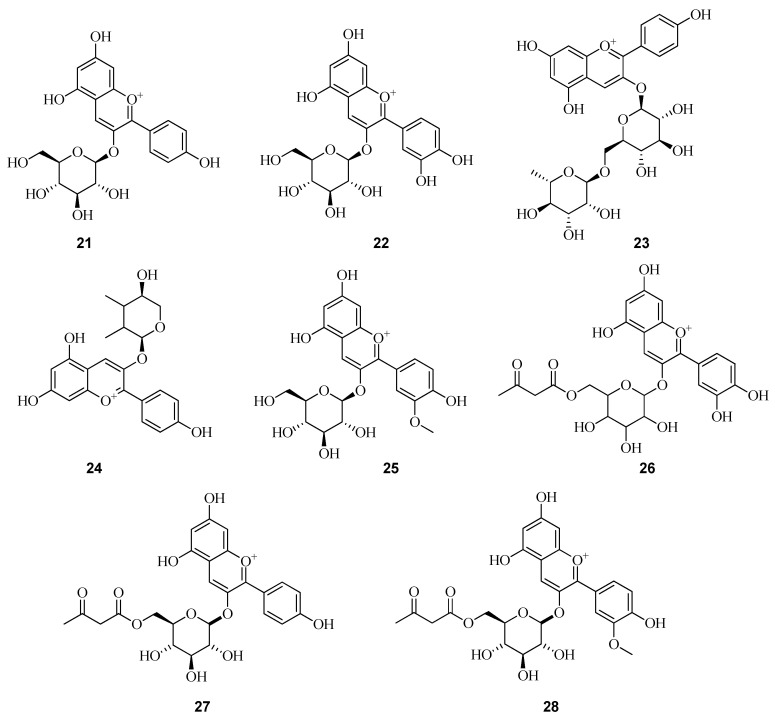
Anthocyanins identified in *Fragaria vesca* and *Fragaria viridis.* Part 1.

The content and composition of anthocyanins vary among different cultivars of *F. vesca*, yet the predominant anthocyanins typically include pelargonidin 3-O-glucoside (**21**), cyanidin 3-O-glucoside (**22**), and pelargonidin 3-O-rutinoside (**23**). For example, a hydromethanolic extract of wild *F. vesca* fruits collected in the northeast region of Portugal revealed six anthocyanins: cyanidin 3-O-glucoside (**22**), pelargonidin 3-O-glucoside (**21**), peonidin 3-O-glucoside (**25**), cyanidin 3-O-malonylglucoside (**26**), pelargonidin 3-O-malonylglucoside (**27**), and peonidin 3-O-malonylglucoside (**28**). Among these, pelargonidin 3-O-glucoside (**21**) was identified as the major anthocyanin (Figure 5). Notably, malonylated derivatives were reported in *F. vesca* for the first time [50].

Malonylated anthocyanin derivatives were identified in the fruits of two diploid inbred lines of *F. vesca* f. Semperflorens-Ruegen F7-4 (a red-fruited genotype) and YW5AF7 (a yellow-fruited genotype) using ultra-high-performance liquid chromatography coupled with high-resolution tandem mass spectrometry (UHPLC-HRMS(n)) [51]. In Ruegen F7-4 fruits, malonyl derivatives (**26–28**) were detected alongside cyanidin 3-O-glucoside (**22**), peonidin 3-O-glucoside (**25**), and pelargonidin 3-O-malonylglucoside (**27**). The authors report that both peonidin 3-O-glucoside (**25**) and peonidin 3-O-malonylglucoside (**28**) were first identified in *F. vesca* var. Ruegen F7-4. In contrast, mature fruits of the YW5AF7 line exclusively contained pelargonidin 3-O-glucoside (**21**), with no malonylated anthocyanins detected.

The chemical composition of fruit extracts from *F. vesca* collected in the Campania region of Southern Italy was investigated by a group of Italian researchers. Both wild and cultivated specimens were harvested from geographically distinct locations. The extracts were subjected to liquid chromatography coupled with high-resolution mass spectrometry (LC-ESI-Orbitrap-MS) to enable comprehensive metabolite profiling. A total of nine anthocyanins were detected and structurally characterized in the methanolic extracts of both wild and cultivated strawberries of various origins. These included cyanidin 3-O-glucoside (**22**), pelargonidin 3-O-glucoside (**21**), pelargonidin 3-O-rutinoside (**23**), peonidin 3-O-glucoside (**25**), cyanidin 3-O-malonylglucoside (**26**), pelargonidin 3-O-malonylglucoside (**27**), peonidin 3-O-malonylglucoside (**28**), delphinidin 3-O-glucoside (**29**), and delphinidin 3-O-malonylglucoside (**30**). The relative content of anthocyanins was found to vary depending on the geographic origin of the fruit samples [52].

In strawberry fruits, anthocyanins account for approximately 58.1% to 81.0% of the total phenolic content. Numerous studies showed that the major anthocyanin in strawberries is pelargonidin 3-O-glucoside (**21**). It accounts for approximately 60% to 95% of the total anthocyanin content. The second most abundant anthocyanin is pelargonidin 3-O-malonylglucoside (**27**), whose levels may range from 0% to 33.5%, depending on the genotype. The anthocyanin content in strawberries is strongly influenced by the genetic background of the specific cultivar or form. It is well established that anthocyanins are key determinants of fruit coloration [53]. Therefore, visual assessment can serve as a preliminary method for estimating anthocyanin levels in analyzed samples. Anthocyanins are synthesized from their aglycone precursors, anthocyanidins such as pelargonidin, cyanidin, and delphinidin, through conjugation with glycosyl, acyl, and methyl groups in various combinations (Table 2). This structural diversification resulting from such modifications underlies the extensive color variability observed in flowers, fruits, and related plant tissues [54].

**Figure 6:**
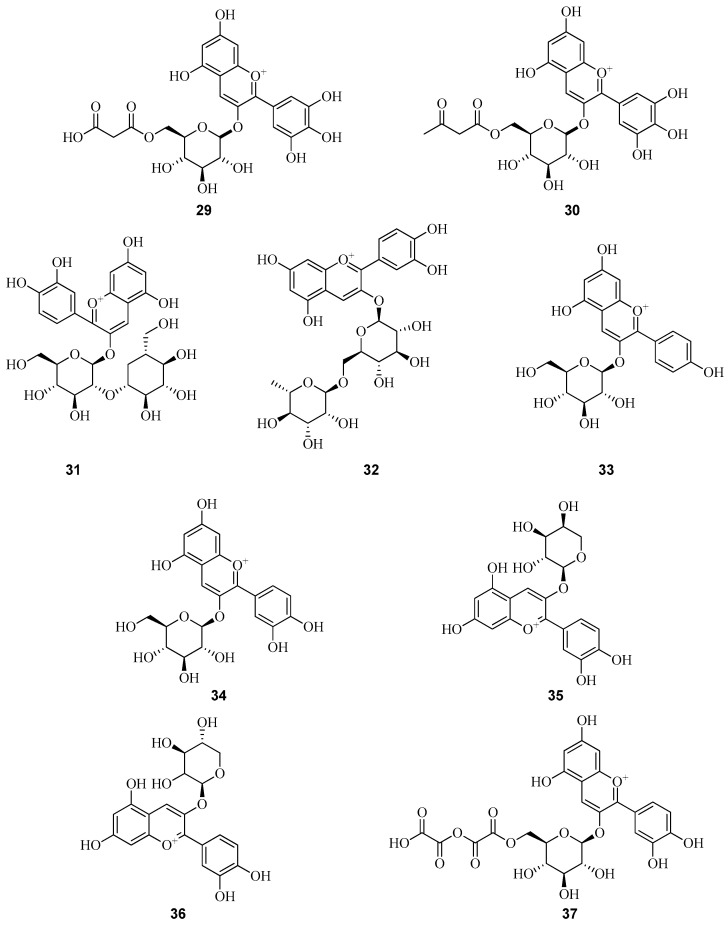
Anthocyanins identified in *Fragaria vesca* and *Fragaria viridis.* Part 2.

In the study conducted by Olennikov et al. [55], the metabolite profile of *F. viridis* fruits was analyzed at three distinct ripening stages. The characterization was performed using high-performance liquid chromatography (HPLC) with a photodiode array detector, coupled to electrospray ionization triple quadrupole mass spectrometry (ESI-QqQ-MS). Prior to the main analysis, extraction protocols for fresh fruit samples were systematically optimized. Various solvents, including methanol, ethanol, isopropanol, water, and acetone, were evaluated. In addition, different solvent-to-material ratios, temperature conditions (ranging from 20 °C to 90 °C), and extraction techniques (ultrasound-assisted, microwave-assisted, and water bath extraction) were tested. As a result of this preliminary optimization, the final extraction procedure employed 100% methanol at a solvent-to-material ratio of 1:1. This was followed by a 5 min homogenization step and ultrasonic treatment for 30 min at 45 °C. Fruit samples of *F. viridis* were collected in the Republic of Sakha (Yakutia) at three distinct stages of ripening. Analysis revealed the presence of both cyanidin and pelargonidin derivatives. Specifically, cyanidin-based anthocyanins included cyanidin 3-O-sophoroside (**31**), cyanidin 3-O-rutinoside (**32**), and cyanidin 3-O-glucoside (**22**). Pelargonidin derivatives comprised pelargonidin 3-O-rutinoside (**23**) and pelargonidin 3-O-glucoside (**33**) (Figure 6). Compound identification was based on characteristic ultraviolet irradiation (UV)-visible absorption spectra-ranging from 525 to 535 nm for cyanidins, and from 498 to 505 nm for pelargonidins. Additional confirmation was provided by aglycone fragmentation patterns in mass spectrometry, with m/z 285 for cyanidins and m/z 269 for pelargonidins. Identification was further supported by comparison with authentic reference standards. Notably, this study reports for the first time the detection of p-coumaroyl anthocyanin esters in *Fragaria* fruits.

In the methanolic extract of *F. vesca* berries collected in central Portugal, six cyanidin-based anthocyanins (**22, 34–38**) were identified using HPLC coupled to a LCQ Deca XP MAX mass spectrometer (Thermo Electron, Bremen, Germany) equipped with an ESI source operating in positive ion mode. Quantification of these compounds was performed at an absorbance wavelength of 530 nm. Among the identified anthocyanins, cyanidin 3-O-glucoside (**22**) was found to be the most abundant, with a concentration of 1945.3 ± 98.5 mg/kg [56].

Thus, anthocyanins in *F. vesca* and *F. viridis* represent a major group of polyphenolic compounds. They are responsible for the characteristic red to purple pigmentation of the berries and are known to exhibit significant biological activity. Several anthocyanidin aglycones have been identified in both *F. vesca* and *F. viridis*, with cyanidin and pelargonidin derivatives predominating. The principal anthocyanins detected in strawberry fruits include cyanidin 3-O-glucoside; pelargonidin 3-O-glucoside; and, to a lesser extent, delphinidin 3-O-glucoside. Anthocyanin biosynthesis in *Fragaria* species is regulated by genes associated with the phenylpropanoid pathway. This process is influenced by ripening stage, environmental conditions, light exposure, and stress factors. Peak anthocyanin concentrations are typically observed in fully ripe fruits, as well as in response to UV or mechanical damage. From a pharmacognostic and nutraceutical perspective, *Fragaria*-derived anthocyanins are of considerable interest as promising compounds for the development of antioxidant and anti-aging therapeutics. Additionally, they may serve as biochemical markers of fruit maturity.

Despite the promising potential of anthocyanins as components in food, cosmetic, and pharmaceutical formulations, their broad industrial application remains limited by several key factors. Effective utilization requires high purity and retention of biological activity throughout production and storage. However, anthocyanins are inherently labile compounds. They are prone to structural degradation, metal ion complexation (with K, Mg, Ca), oxidative reactions, and polymerization. The presence of structurally similar flavonoids further complicates the standardization of plant raw materials. In addition, the economic feasibility of large-scale anthocyanin production must be carefully considered. Therefore, targeted selection of *Fragaria* species and genotypes with high anthocyanin content is essential for the development of cultivars suitable for industrial cultivation.

### 6.2. Flavonols in Fragaria vesca and Fragaria viridis

Flavonols represent one of the key classes of flavonoids widely distributed in plants, including strawberries *(Fragaria* spp.). These compounds exhibit a broad spectrum of biological activities. They are known for their antioxidant, anti-inflammatory, antimicrobial, and vasoprotective properties. Flavonols possess the ability to scavenge free radicals, inhibit lipid peroxidation, and modulate the activity of various enzymes involved in cellular defense mechanisms. Recent evidence also indicates that flavonols may exert neuroprotective effects by supporting cognitive function and lowering the risk of neurodegenerative diseases.

The major flavonols identified in strawberry leaves and fruits exhibit strong antioxidant activity. They play a critical role in protecting plant tissues from oxidative stress. Their concentration can vary significantly depending on species and cultivar, fruit ripening stage, environmental growing conditions, and the extraction methods employed. Due to their bioactive properties, flavonols from *Fragaria* spp. are of considerable interest in pharmacology and nutraceutical research. They hold significant potential as natural antioxidants. These compounds may contribute to the development of both preventive and therapeutic agents. Such agents could target a range of disorders, including cardiovascular and neurodegenerative diseases.

Quercetin (**38**) is a major bioactive constituent in *Fragaria* species and serves as a pharmacologically active ingredient in numerous therapeutic formulations [57]. Among its most significant pharmacological properties are antioxidant, antidiabetic, anticancer, antitumor, anti-inflammatory, antiallergic, antihypertensive, and antidepressant activities. Myricetin (**39**) and kaempferol (**40**), along with their glycosylated derivatives, are also prominent flavonols found in *Fragaria* spp. These compounds exhibit high pharmacological potential and contribute to the overall therapeutic value of the plant matrix [58,59].

The qualitative composition and quantitative determination of flavonols in leaf extracts of *F. vesca* collected in Croatia were analyzed under optimized extraction conditions using ultrasound-assisted and microwave-assisted methods. Flavonol profiling was performed by UPLC coupled with tandem mass spectrometry (MS-MS). The following flavonols were identified: quercetin (**38**), myricetin (**39**), kaempferol (**40**), isorhamnetin (**41**), quercetin 3-O-glucoside (**42**), rutin (**43**), quercetin-3-glucuronide (**44**), quercetin-3-rhamnoside (**45**), quercetin-3-pentoside (**46**), quercetin-acetyl-hexoside (**47**), myricetin-3-O-rhamnoside (**48**), myricetin-3-O-galactoside (**49**), myricetin-3-O-arabinoside (**50**), kaempferol-3-rutinoside (**51**), kaempferol-3-glucuronide (**52**), kaempferol-3-O-hexoside (**53**), kaempferol-3-O-pentoside (**54**), kaempferol-pentosyl-hexoside (**55**), kaempferol-acetyl-hexoside (**56**), kaempferol-acetyl-rutinoside (**57**), isorhamnetin-3-rhamnoside (**58**), and isorhamnetin-3-hexoside (**59**) [24]. This study represents the first comprehensive characterization of diverse flavonol derivatives in *F. vesca*, with eight kaempferol derivatives and nine quercetin derivatives identified. The content of major flavonols, rutin, quercetin, kaempferol, and their glycosylated forms, was substantially higher in ultrasound-assisted extracts compared to those obtained via microwave-assisted extraction.

In contrast, a study by Portuguese researchers, employing HPLC at 280 nm following maceration of a 50% aqueous-ethanol extract of *F. vesca* (1:10, *w*/*v*), identified only four quercetin and kaempferol glycosides. However, their concentrations were reported to be very low [23].

In the fruits of cultivated *F. vesca* of cultivars Ruegen F7-4 and YW5AF7, only trace amounts of quercetin 3-O-glucoside (**42**), quercetin-acetyl-hexoside (**47**), and two kaempferol 3-O-acetyl-hexoside derivatives were identified [51].

In fruits of Italian *F. vesca*, the following flavonols were detected: quercetin rhamnoside (**45**), isoquercitrin (**60**), quercetin glucuronide (**44**), kaempferol glucuronide (**52**), and kaempferol coumaroyl hexoside (**61**). Among these, quercetin glucuronide (**44**) was found at comparatively high concentrations [52].

In the study conducted by D’Urso et al. [60], bioactive compounds in the leaves of *F. vesca* collected in Italy were investigated using metabolomics approaches based on liquid chromatography coupled with mass spectrometry. This analytical strategy enabled the detection of a broad spectrum of polyphenolic compounds, including flavonoids, phenolic acids, and tannins. The principal antioxidant constituents were identified as quercetin (**38**), kaempferol (**40**), and ellagic acid derivatives. However, precise quantitative data for these individual compounds were not reported.

Bagdonaitea et al. quantified the levels of rutin (**43**) and quercetin 3-O-glucoside (**42**) in the fruits and leaves of *F. vesca* and *F. viridis*, cultivated under identical conditions in Lithuania, using HPLC. Among the analyzed samples, *F. vesca* fruits exhibited the highest concentrations: 1.38 ± 0.19 mg/g for (**43**) and 0.69 ± 0.10 mg/g for (**42**). *F. vesca* demonstrated the highest overall content of the targeted phenolic compounds among the species studied. This finding highlights its potential as a valuable source of biologically active substances [61].

**Figure 7:**
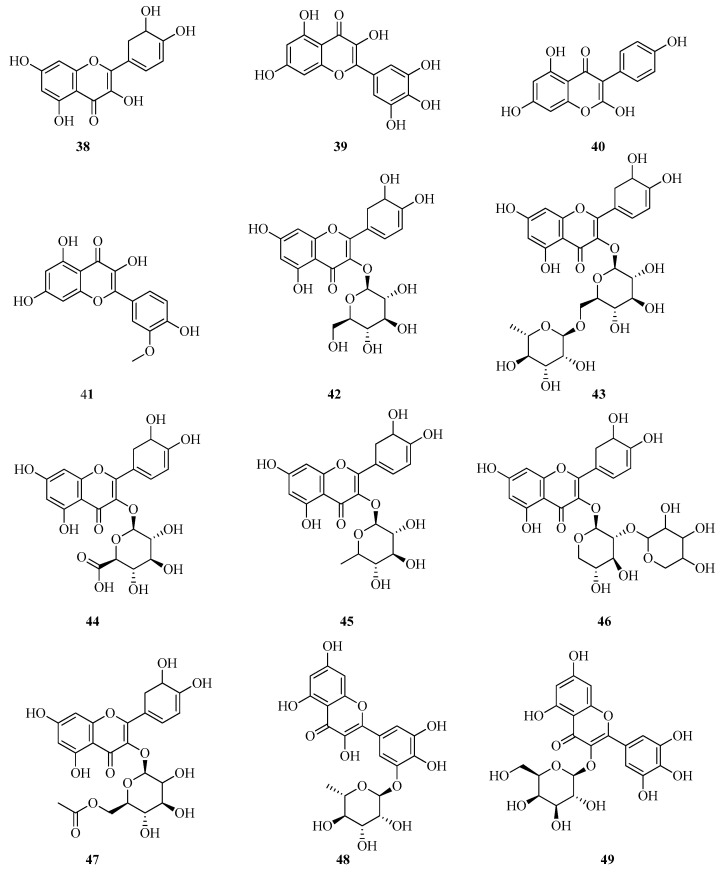
Flavonols in *Fragaria vesca* and *Fragaria viridis*. Part 1.

In the study carried out by Stoenescu et al. [62], Romanian researchers conducted a quantitative analysis of flavonoid content in various anatomical parts of *F. viridis*. Phenolic compounds were extracted using 100% methanol. The analysis was performed on an UltiMate 3000 XRS UHPLC system equipped with a UV–VIS diode array detector, with detection set at 278 nm. Rutin (**43**) was identified as the predominant flavonoid in the inflorescences, with a concentration of 242.36 ± 14.20 mg/100 g. Myricetin (**39**) was measured at 66.98 ± 2.15 mg/100 g (Figure 7). In the fruits, rutin was present at 7.50 ± 0.03 mg/100 g, whereas myricetin was not detected.

**Figure 8:**
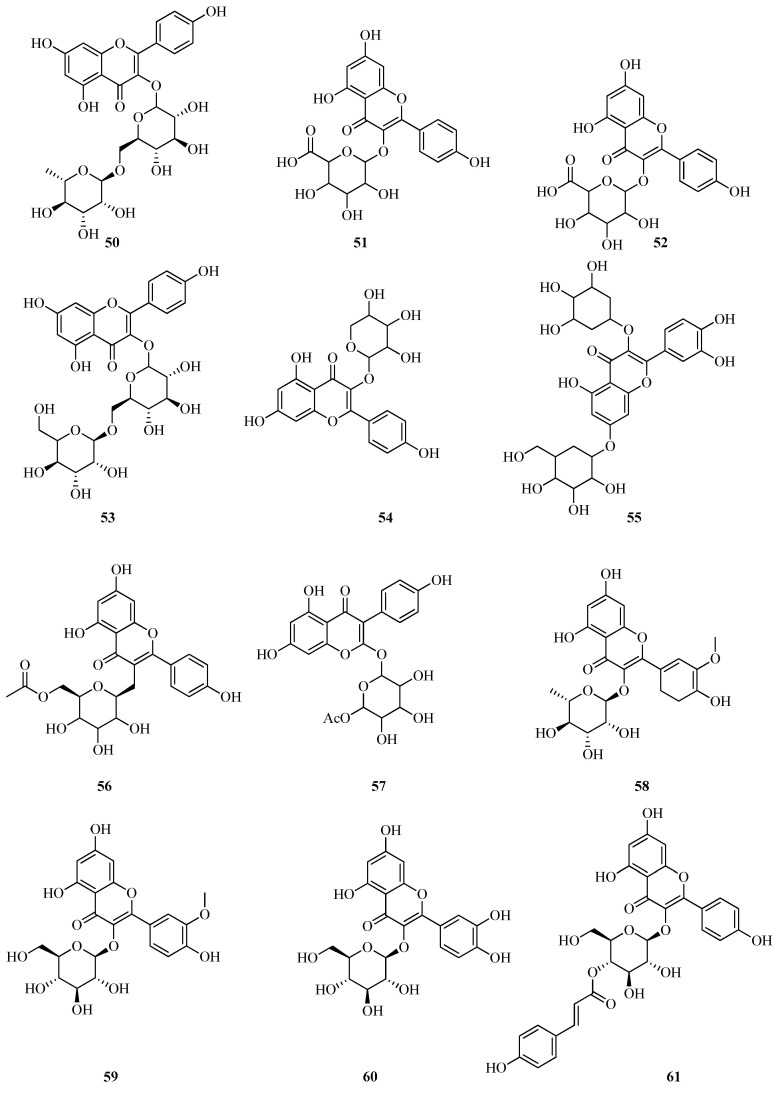
Flavonols in *Fragaria vesca* and *Fragaria viridis*. Part 2.

A detailed analysis of flavonols in the fruits of *F. viridis* at different stages of ripening and during storage was conducted by Yildiz et al. [1]. Using HPLC-PAD-ESI-tQ-MS, flavonols were identified based on their UV spectral profiles, with absorption maxima at 256/268/360 nm for quercetin derivatives and 265/343 nm for kaempferol derivatives. In this study, 34 flavonols were identified in the fruits of *F. viridis*, including 2 aglycones, quercetin and kaempferol, along with 32 glycosidic derivatives, which included both non-acylated and acylated fragments linked to sugar moieties. The total flavonol content in *F. viridis* fruits varied during the ripening process, with unripe fruits containing 1.24 mg/g, and mature fruits showing a decrease to 1.01 mg/g. The total content of quercetin (**38**) and its derivatives ranged from 0.66 to 0.82 mg/g, while kaempferol (**40**) and its derivatives were present at levels of 0.35 to 0.42 mg/g. During the storage of mature *F. viridis* fruits, the following changes were observed: at 4 °C (7 days), the rutin (**43**) content decreased by 16.7% (from 0.24 to 0.20 mg/g). At 20 °C (3 days), a 25% reduction in rutin (**43**) content was noted (from 0.24 to 0.18 mg/g), accompanied by an increase in quercetin 3-O-glucoside (**42**) and quercetin 3-O-glucuronide (**44**) (Figure 8). *F. viridis* fruits are a rich source of flavonols, particularly quercetin derivatives. The highest concentrations of these compounds were observed in unripe fruits, gradually decreasing as the fruits ripened. Storage at low temperatures helps preserve flavonols, which is crucial for maintaining the antioxidant activity of the fruits [1].

Thus, the leaves of *F. vesca* represent a rich source of antioxidants and can be considered a promising raw material for the development of natural medicines and functional foods. The flavonols in the leaves of *F. vesca* are primarily quercetin (**38**) and kaempferol (**40**). As for the glycosidic forms of flavonols, researchers from Commonwealth of Independent States countries have reported that the dominant compound in *F. vesca* leaves is the quercetin glycoside rutin (43). This compound may account for up to 3.2% of the total flavonoid content in the leaves [63,64,65,66,67]. Several international studies report either trace amounts of rutin (**43**) or none at all. Other quercetin derivatives identified include isoquercetin (**60**) and quercetin 3-O-glucoside (**42**).

### 6.3. Flavan-3-ols and Flavan-3,4-diols in Fragaria vesca and Fragaria viridis

Flavan-3-ols constitute a subclass of flavonoids, encompassing both catechins and proanthocyanidins. These compounds belong specifically to the flavanol subgroup within flavonoids. Structurally, flavan-3-ols are characterized by a three-ring backbone comprising rings A, B, and C. Their activity is modulated by the number and position of hydroxyl groups on these rings. Notably, flavan-3-ols lack an oxo group at the C4 position of the C ring. This feature distinguishes them from flavanones. In addition, they do not possess a double bond between the C2 and C3 positions. In both *F. vesca* and *F. viridis*, flavan-3-ols play a pivotal role in antioxidative defense and act as protective secondary metabolites. Key representatives of this class include (+)-catechin (compound **63**), (−)-epicatechin (compound **64**), and oligomeric proanthocyanidins, primarily in the form of dimers and trimers.

Flavan-3,4-diols represent a distinct subclass of flavanols, commonly referred to as leucoanthocyanidins. These compounds are recognized as biosynthetic precursors of both anthocyanidins and proanthocyanidins. Structurally, they are colorless due to the absence of conjugated double bonds in the chromophore region. Flavan-3,4-diols differ from flavan-3-ols in that they possess two hydroxyl groups located at the C3 and C4 positions of the flavan backbone. This unique configuration confers distinct chemical reactivity. They are inherently unstable and readily undergo oxidative transformation to yield anthocyanidins. Alternatively, they may participate in polymerization reactions, leading to the formation of proanthocyanidins. These compounds are naturally present in *Fragaria* species, including *F. vesca* and *F. viridis*, with the highest concentrations typically observed in immature fruits and foliage.

**Figure 9:**
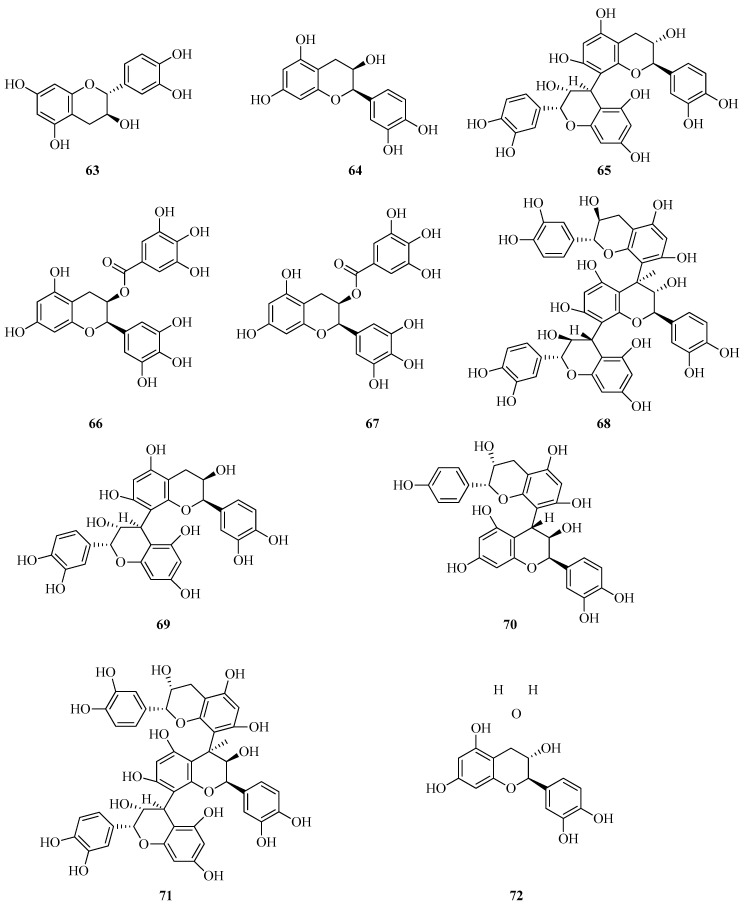
Flavan-3-ols identified in *Fragaria vesca* and *Fragaria viridis*.

Leaf material of *F. vesca*, collected during the summer of 2022 in the Dalmatia region of Croatia, was subjected to ultrasound-assisted cavitation and microwave-assisted extraction techniques. Among the isolated phenolic constituents, (−)-epicatechin (**64**) was detected at a relatively high concentration of 100.29 ± 2.84 mg per 100 g of dry weight in the ultrasound-assisted extracts. Even more pronounced was the accumulation of procyanidin B1 (**65**), quantified at 332.26 ± 9.40 mg/100 g under the same extraction conditions. Other identified compounds included epigallocatechin gallate (**66**), epicatechin gallate (**67**), a procyanidin trimer (**68**), and procyanidin B2 (**69**) (Figure 9). However, these were present only in trace or minor quantities.

In a study D’Urso et al. [52], a total of 39 metabolites were identified in methanolic extracts of *F. vesca* leaves using LC-ESI-Orbitrap-MS. Among the compounds detected were procyanidin B1 (**65**), (epi)afzelechin-(epi)catechin dimers (**70**), procyanidin C1 (**71**), and procyanidin B2 (**69**). In the same study, the authors also investigated the metabolite composition of *F. vesca* leaves preserved via immediate flash-freezing in liquid nitrogen, followed by lyophilization. For extraction, 500 mg of freeze-dried leaf material was treated with 10 mL of 70:30 ethanol–water solution. The mixture was subjected to 15 min of ultrasonic treatment and subsequently centrifuged at 1750 rpm for 15 min. A total of 27 metabolites were identified using LC-ESI/LTQ-Orbitrap-MS. These were predominantly classified as organic acids, flavonoids, catechin derivatives, and catechin oligomers [60].

In ultrasound-assisted leaf extracts of *F. viridis*, both (+)-catechin hydrate (**72**) and (−)-epicatechin (**64**) were detected at concentrations of 311.76 ± 21.35 mg/100 g and 778.11 ± 15.10 mg/100 g dry weight, respectively [62].

In *F. vesca*, multiple flavan-3-ols were identified, including (+)-catechin (**63**), B-type proanthocyanidin dimers, B-type trimers, and B-type tetramers. The identification was supported by HRMS^n^ data, UV spectral characteristics, and comparison with literature references [51].

Catechin (**63**) was isolated from an aqueous extract of *F. vesca* leaves. The extract was purified using SPE C18 (Supelco, USA). Water was employed as the washing solvent, while methanol was used for elution of the target compound from the column. Final isolation was performed using reversed-phase HPLC coupled with a diode array detector [66].

Catechin derivatives belong to the broader class of flavan-3-ols and were detected both as monomeric units and as structural subunits within proanthocyanidin oligomers. These ranged from dimers to higher-order polymers. In a study conducted by researchers [68], two chromatographic peaks with characteristic galloyl-related UV absorption features were observed. These peaks appeared after acid hydrolysis of *F. vesca* extracts obtained from plants cultivated in Bolivia.

Water-soluble procyanidins obtained through the fermentation of a tannin-rich extract from *F. vesca* roots were analyzed using HPLC. Three B-type procyanidin dimers, namely, procyanidins B1, B2, and B5, were identified and quantified. In addition, two flavan-3-ol monomers, (+)-catechin (**63**) and (−)-epicatechin (**64**), were detected. These procyanidins demonstrated both antibacterial activity and pronounced angioprotective properties [69].

Furthermore, in a water-methanol extract of *F. vesca* fruits collected in the northeastern region of Portugal, (+)-catechin (**63**) was detected in appreciable quantities using HPLC-DAD-MS/ESI analysis [23].

In a recent study [70], the accumulation of polyphenols and the expression of genes involved in their biosynthesis were investigated in fruits of two *F. vesca* genotypes: the red-fruited “Baron Solemacher” (BS) and the white-fruited “Pineapple Crush” (PC). The analysis encompassed four developmental stages: early green (G1), late green (G2), turning (T), and fully ripe (R). At the ripe stage (R), “Baron Solemacher” exhibited a markedly higher flavan-3-ol content (417 mg/100 g fresh weight) compared to “Pineapple Crush” (210 mg/100 g). These findings highlight distinct metabolic pathway activities between the two genotypes. Catechin, a key flavan-3-ol, is synthesized via the phenylpropanoid–flavonoid biosynthetic pathway. In “Baron Solemacher”, the expression of several structural genes associated with this pathway, PAL1, CHS, F3’H, DFR, ANS, and UFGT1, was significantly upregulated at the ripe stage. Crucially, the transcription factor MYB10, known to regulate the expression of these genes, was also expressed at substantially higher levels in the red-fruited genotype during fruit ripening. In contrast, the white-fruited “Pineapple Crush” showed reduced expression of MYB10 and the aforementioned structural genes, resulting in lower accumulation of flavan-3-ols, including catechin. These data suggest that the differential expression of flavonoid biosynthetic genes directly influences catechin (63) accumulation in *F. vesca* fruits. This regulation is genotype-dependent and is particularly controlled at the transcriptional level by the MYB10 transcription factor.

Blanch et al. [71] comprehensively examined the impact of high-concentration CO_2_ treatment on catechin and proanthocyanidin content in *F. vesca* berries. Postharvest treatment of fruits was conducted under elevated CO_2_ atmospheres (20% and 40%) at low temperatures to evaluate its effect on flavonoid biosynthesis, including catechins, proanthocyanidins, and anthocyanins. The CO_2_-treated berries exhibited a notable increase in the levels of proanthocyanidins B1 and B3. Flavonoid profiling was conducted using quadrupole time-of-flight mass spectrometry (Q-TOF). Quantitative analysis was performed by high-performance liquid chromatography (HPLC) coupled with quadrupole mass spectrometry. The observed increase in catechin concentrations following CO_2_ treatment may contribute to reduced fungal spoilage. This effect is likely attributable to the well-documented antimicrobial properties of catechins.

Quantitative data on catechin and proanthocyanidin content in *F. viridis* fruits at different ripening stages have been reported [55]. Fresh fruit samples (100 g) were extracted twice with 100 mL of methanol using an ultrasonic water bath at 50 °C for 30 min per extraction. Compound identification and quantification were performed using HPLC equipped with a photodiode array detector and a triple quadrupole mass spectrometer with ESI. Calibration was conducted using authentic standards, including catechin and proanthocyanidin references. The total content of catechins and proanthocyanidins was found to decrease progressively during fruit maturation. In unripe fruits, the concentration reached 0.29 mg/g fresh weight, decreasing to 0.17 mg/g in semi-ripe fruits and further to 0.09 mg/g in fully ripe fruits.

Catechins and proanthocyanidins are key representatives of the flavanol subclass, playing a central role in antioxidant capacity, flavor development, and defense against biotic stressors in *Fragaria* species. *F. vesca* is characterized by a more active phenolic metabolism, particularly under stress conditions such as postharvest CO_2_ treatment. These stressors induce the accumulation of catechins, including (−)-epicatechin, and B-type proanthocyanidin dimers such as B1 and B3. This reflects a high degree of plasticity in the phenolic profile and suggests that flavonoid biosynthesis in *F. vesca* can be modulated by external environmental cues. These compounds contribute to defense responses and may enhance resistance to fungal pathogens. In contrast, *F. viridis* shows a gradual decline in catechin and proanthocyanidin levels (e.g., B2 and C2) during natural ripening. This trend reflects a phenolic shift toward anthocyanin dominance as fruit maturity progresses. Peak levels of catechins and proanthocyanidins in *F. viridis* are observed during the early developmental stages. These compounds subsequently undergo metabolic transformation or degradation as the fruit matures. This indicates a limited capacity for their sustained accumulation and a lower phenolic adaptability to environmental stimuli, compared to *F. vesca*.

*F. vesca* exhibits higher biochemical activity and a greater capacity for adaptive accumulation of catechins and proanthocyanidins. In contrast, *F. viridis* is characterized by a more stable yet less flexible flavanol profile. Peak concentrations of these compounds in F. viridis are detected at early developmental stages. These interspecific differences reflect distinct modes of metabolic regulation and may be relevant for the development of functional food products and for optimizing fruit storage strategies.

### 6.4. Phenolic Acids and Their Derivatives in Fragaria vesca and Fragaria viridis

Phenolic acids identified in *F. vesca* and *F. viridis* represent a class of biologically active compounds with strong antioxidant, anti-inflammatory, and antimicrobial properties. These metabolites participate in plant defense mechanisms by enhancing resilience to environmental stressors. In addition, they contribute significantly to the flavor, aroma, and nutritional value of both fruit and leaf tissues.

Due to their chemical structure, which includes hydroxyl and carboxyl functional groups, phenolic acids exhibit high chemical reactivity. They actively participate in modulating redox processes within plant and animal cells. These compounds are extensively studied for their potential applications in pharmacology, cosmetology, and the food industry. Their pronounced antioxidant capacity contributes to cellular protection against oxidative stress. Consequently, this reduces the risk of developing chronic diseases.

Of particular interest are hydroxycinnamic and hydroxybenzoic acids identified in the leaves of *Fragaria* species. These compounds exhibit not only strong antioxidant properties but also notable antimicrobial activity. The composition and concentration of phenolic acids can be influenced by various extraction techniques. This offers promising opportunities for their targeted application in biomedical and technological fields. Phenolic acids represent important phytochemical constituents in both *F. vesca* and *F. viridis*. Notably, the wild genotype, *F. vesca*, tends to accumulate higher levels of these compounds [48].

**Figure 10:**
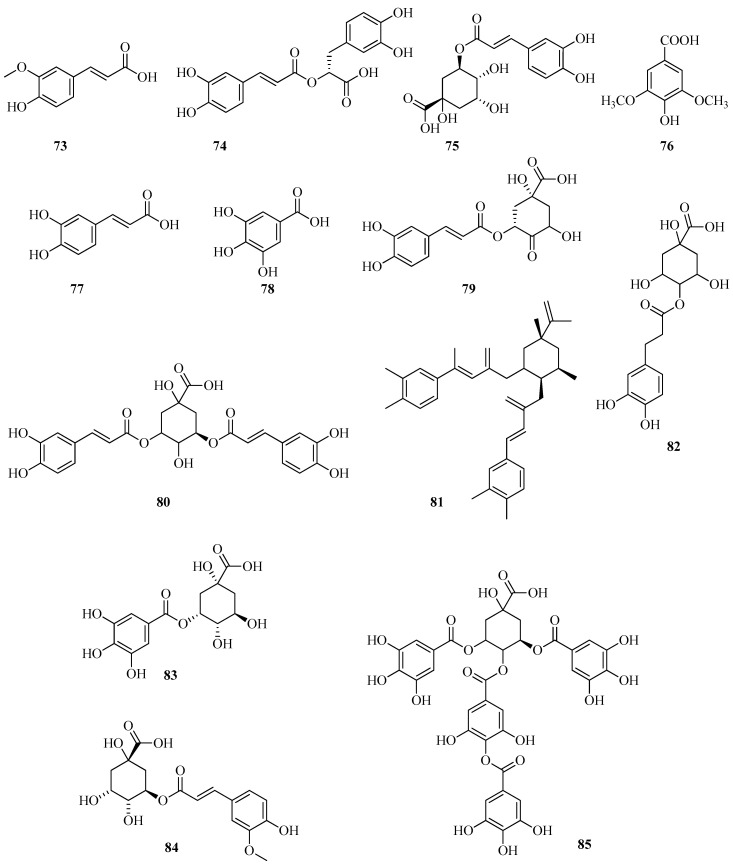
Phenolic acids identified in *Fragaria vesca* and *Fragaria viridis*: Part 1.

UPLC/MS-MS was employed to profile the phenolic composition of *F. vesca* leaf extracts. Several phenolic acids were identified through comparison with authentic standards, including ferulic (**73**), rosmarinic (**74**), chlorogenic (**75**), syringic (**76**), caffeic (**77**), and gallic acids (**78**). Based on previously established fragmentation patterns [72], a number of additional derivatives were tentatively identified (Figure 10). These included 3-p-caffeoylquinic (**79**), 3,5-di-O-caffeoylquinic (**80**), 4,5-di-O-caffeoylquinic (**81**), 4-O-caffeoylquinic (**82**), 5-O-galloylquinic (**83**), 3-O-feruloylquinic (**84**), and 3,5-digalloylquinic acids (**85**). The presence of 4-O-caffeoylquinic acid (**82**) confirms earlier findings [60], whereas the remaining derivatives are reported here for the first time [24].

Microwave-assisted extraction at 80 °C increased the total yield of phenolic acids by 40% compared to ultrasound-assisted extraction. Although both methods resulted in extracts dominated by four major acids, p-hydroxybenzoic (**86**), gallic (**78**), 5-O-galloylquinic (**83**), and chlorogenic acid (**75**), their relative distributions differed depending on the extraction technique. Microwave extraction was 109% more efficient in recovering 4-O-caffeoylquinic acid (**82**) and showed significantly enhanced extraction of phenolics containing varying numbers of hydroxyl groups. In contrast, ultrasound-assisted extraction favored the recovery of ferulic (**73**) and syringic (**76**) acids [24].

**Figure 11:**
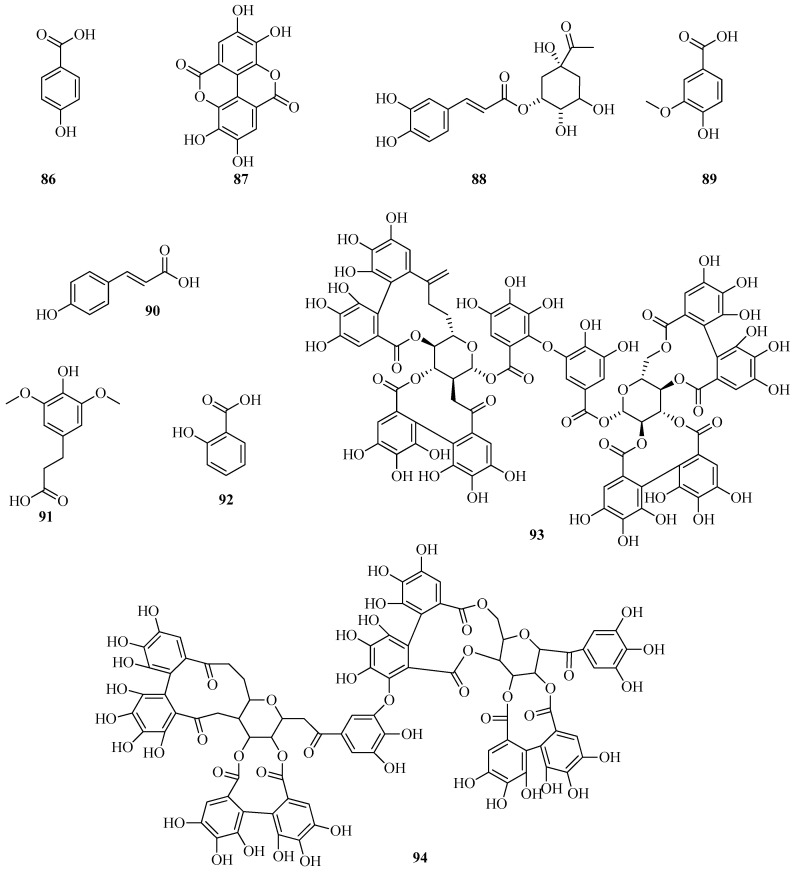
Phenolic acids identified in *Fragaria vesca* and *Fragaria viridis*: Part 2.

*Fragaria* species typically contain a broad spectrum of low-molecular-weight phenolic metabolites. However, the total phenolic content is generally lower compared to that of other commonly consumed berries, such as blueberries, bilberries, and raspberries. According to a recent review [73], the phenolic content in *F. vesca* varies within the following ranges (mg per 100 g fresh weight): ellagic acid (**87**), 9.7–34.5; gallic acid (**78**), 5.6–44.0; and ferulic acid (**73**), 1.5–5.1 (Figure 11). Notably, the highest concentration of ellagic acid (**87**) was observed in green-stage fruits, reaching 351.96 mg/100 g dry weight.

Using HPLC-UV analysis, Stoenescu et al. identified a range of phenolic acids in methanolic extracts obtained via ultrasound-assisted cavitation. The compounds detected included gallic acid (**78**), neochlorogenic acid (**88**), chlorogenic acid (**75**), vanillic acid (**89**), syringic acid (**76**), p-coumaric acid (**90**), ferulic acid (**73**), sinapic acid (**91**), salicylic acid (**92**), and ellagic acid (**87**). Among these, gallic acid (**78**) was the most abundant, particularly in the green-stage fruits of *F. vesca*, where its concentration reached 122.02 mg/100 g dry weight. Syringic acid (**76**) was found in only one sample type, green leaves and strawberry flowers, at concentrations of 131.01 mg/100 g DW and 29.05 mg/100 g DW, respectively [62].

*F. vesca* leaves represent a rich and largely underutilized source of biologically active compounds with well-documented health-promoting properties. Their chemical composition has been comprehensively characterized using UPLC–ESI-MS/MS. Among the identified phenolic constituents, neochlorogenic acid (**88**) was quantified at a concentration of 5.05 mg/g dry weight [74].

Ellagitannins are complex esters of ellagic acid and carbohydrates, most commonly glucose. They are formed by the oxidative coupling of two gallic acid residues, resulting in the creation of a bixicyclohexenone system. Upon hydrolysis, this structure releases ellagic acid. In other words, ellagitannins are tannins that contain ellagic acid in a bound form, which is released upon hydrolysis. These compounds are widely distributed in plants and exhibit pronounced antioxidant and anti-inflammatory activities [75]. It has been found that extracts from the leaves and fruits of *F. vesca* contain ellagic acid (**78**) in sufficient amounts compared to the flowers [62].

**Figure 12:**
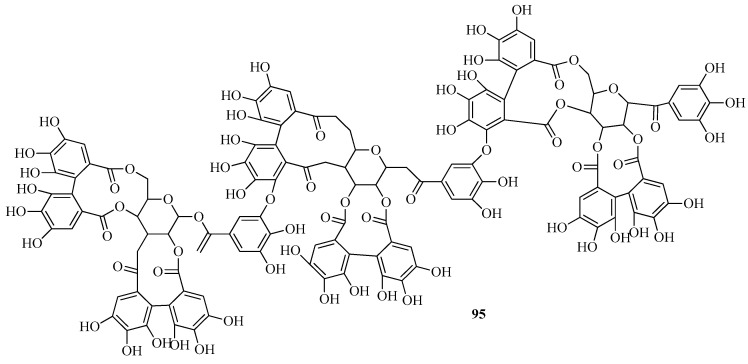
Ellagitannins from *Fragaria vesca* and *Fragaria viridis*.

In a study performed by Vrhovsek et al. [76], agrimoniin (**93**) was isolated from the fruits of *F. vesca*, and its structure was determined through spectral data. It was also established that agrimoniin is the primary ellagitannin present in the fruits of *F. vesca*. Additionally, sanguin H-6 (**94**) and lambertianin C (**95**) were identified (Figure 12).

Ellagitannins and other phenolic compounds in the fruits of two diploid inbred lines of *F. vesca f.* semperflorens—Ruegen F7-4 and YW5AF7, were characterized using UHPLC-HRMS(n). Identified in minor quantities were methylpentoside ellagic acid, methylpentoside methyl ellagic acid, 3-O-acetyl-hexoside methyl ellagic acid, 3-O-pentoside methyl ellagic acid, galloyl-bis-HHDP-glucose, and trigalloylglucose [51].

Gallic acid (**78**) and ellagic acid (**87**) were identified from samples of *F. vesca* collected at altitudes ranging from 2650 to 3300 m above sea level in Bolivia [68].

Various fractions were obtained from the aqueous-ethanolic extract of *F. vesca* leaves by gel chromatography using a Sephadex LH-20 column (Sigma-Aldrich). Fractionation was achieved through sequential elution with 50% aqueous methanol (2 L), 75% aqueous methanol (2.5 L), and 70% aqueous acetone (1 L). The entire fractionation process was monitored by HPLC, which yielded eight major fractions. The fraction enriched in ellagitannins was profiled at 280 nm. Twelve compounds within this fraction were tentatively identified as ellagitannins using HPLC-PDA-ESI/MSn. Identification was based on their characteristic UV and mass spectral features. All detected compounds exhibited a typical UV absorption profile, with a maximum below 270 nm. Most compounds showed an absorption maximum between 248 and 257 nm, along with a minor shoulder at approximately 280–284 nm. Among the identified compounds were ellagic acid (m/z 87), ellagitannin derivatives, galloyl-HHDP-glucose conjugates, and sanguine H-2, among others [77].

*F. vesca* has been recognized as a rich source of ellagitannins and ellagic acid glycosides. Milczarek et al. [78] evaluated the influence of fruit comminution methods on the efficiency of ellagitannin extraction. Specifically, they compared homogenization with cryogenic grinding. In addition, two extraction activation strategies were compared: ultrasonic treatment and mechanical shaking. In the second phase of the study, response surface methodology (RSM) was employed to determine the optimal extraction parameters. Experimental variables included acetone concentration (40%, 60%, and 80%), ultrasound duration (5, 10, and 15 min), and temperature (20, 35, and 50 °C). The resulting extracts were quantitatively analyzed by HPLC with a diode-array detector (DAD), and compound identification was confirmed using mass spectrometry. The comminution step was found to be critical for efficient ellagitannin recovery. On average, extracts from cryogenically milled fruits contained approximately 20% less ellagitannins. This amount is lower compared to extracts obtained from homogenized material. According to RSM analysis, acetone concentration was the most significant factor influencing extraction yield. The highest ellagitannin content was observed at 80% acetone, within the tested range of 40–80%. Neither extraction temperature nor ultrasound duration significantly affected extraction efficiency. Agrimoniin (m/z 93) was identified as the major ellagitannin present in *F. vesca*.

In the fruits of *F. viridis*, ellagic acid (m/z 87), four ellagic acid glycosides, and eighteen distinct ellagitannins were identified using authentic reference standards [55]. These included lambertianin C (m/z 95), sanguin H-10, sanguin H-6, sanguin H-2, and pedunculagin, as well as structurally characterized compounds such as strictinin, castalagin, and casuarictin. Further analysis via MS/MS confirmed the presence of agrimoniin (m/z 93), the ellagitannin fragarian A, and agrimonic acids A and B. Ellagitannins were found to be the predominant class of secondary metabolites in *F. viridis* fruits, with total concentrations ranging from 5.97 to 7.54 mg/g fresh weight. Among the quantified compounds, agrimoniin (1.41–2.63 mg/g) and lambertianin C (1.20–1.86 mg/g) were identified as the major constituents.

Thus, a variety of polyphenolic acids were identified in the fruits of both strawberry species, *F. vesca* and *F. viridis*, including ellagic acid (**87**) and gallic acid (**78**), along with their respective derivatives. Gallic acid (**78**) was found at lower concentrations compared to ellagic acid (**87**). Notably, ellagic acid was detected both in its free form and as part of glycosidic and tannin complexes. It contributes substantially to the antioxidant profile of the berries. It is worth noting that *F. viridis* exhibited a tendency toward slightly higher gallic acid (**78**) content relative to *F. vesca*. This difference may be attributed to variations in the biosynthetic pathways between the two species. Nevertheless, ellagic acid (**87**) and its associated tannins, particularly agrimoniin (**93**), remain the predominant polyphenolic constituents in both species.

### 6.5. Other Compounds in Fragaria vesca and Fragaria viridis

The fruits of *F. vesca* and *F. viridis* are recognized as valuable sources of ascorbic acid (vitamin C). This compound plays a pivotal role in antioxidant defense. Additionally, it contributes to the stabilization of phenolic compounds within plant tissues [79]. The concentration of ascorbic acid can vary depending on environmental conditions, fruit maturity, and genetic factors. Nonetheless, *F. vesca* typically exhibits a slightly higher level of this vitamin compared to *F. viridis*. In addition to ascorbic acid, both species also contain various organic acids, including citric, malic, tartaric, and fumaric acids. Citric acid predominates in the overall acid profile, contributing to the characteristic refreshing flavor of the berries. Malic acid is also present in significant quantities, where it participates in the regulation of acidity and the stabilization of metabolic compounds. The specific ratio of organic acids can serve as a biochemical marker of fruit maturity and cultivar identity. Moreover, it influences both the sensory characteristics and the stability of biologically active compounds, such as polyphenols and vitamins. Taken together, *F. vesca* and *F. viridis* exhibit a favorable acid composition that combines the high ascorbic acid content with a diverse spectrum of organic acids. This contributes significantly to their nutritional quality and functional potential.

Beyond polyphenolic and organic acids, the fruits of *F. vesca* and *F. viridis* contain a wide array of biologically active compounds. These include flavonoids, anthocyanins, sugars, pectins, amino acids, and aromatic constituents. The berries also contain appreciable levels of soluble sugars, namely, glucose, fructose, and sucrose, which contribute to their characteristic sweetness. Additionally, the fruits are rich in dietary fiber, particularly pectins. These compounds are important not only for digestive health but also for the technological functionality of the raw material in food processing.

A variety of triterpenoids and steroids have also been identified in the fruits and other plant parts of *F. vesca* and *F. viridis*. These compounds play essential roles in plant physiological processes and exhibit potential pharmacological activity [27].

Strawberry fruits contain a broad spectrum of mineral elements, including phosphorus (P), calcium (Ca), cobalt (Co), and manganese (Mn). Notably, the iron (Fe) content in strawberry fruits is approximately twice that found in plums. It is also up to 40 times higher than in grapes. This highlights their superior mineral profile in terms of iron content. In addition, the roots and rhizomes of *F. vesca* and *F. viridis* have been shown to accumulate up to 9.4% tannins, which may contribute to both the plant’s defense mechanisms and its pharmacological potential. Quantitative data on the elemental composition of strawberry leaf ash reveal significant levels of macroelements (mg/g): potassium (K)—21.9; calcium (Ca)—14.7; magnesium (Mg)—4.5; and iron (Fe)—0.6. Microelements (µg/g) include manganese (Mn)—0.22; copper (Cu)—0.84; zinc (Zn)—0.9; cobalt (Co)—0.22; molybdenum (Mo)—1.28; chromium (Cr)—0.3; aluminum (Al)—0.29; barium (Ba)—0.81; vanadium (V)—0.9; selenium (Se)—11.0; nickel (Ni)—0.18; strontium (Sr)—0.75; lead (Pb)—0.25; iodine (I)—0.09; bromine (Br)—78.3; and boron (B)—143.2. Among the studied species, *F. vesca* exhibited the highest levels of iron in its mineral composition [80], further reinforcing its nutritional and functional significance.

Polysaccharides have been isolated from the leaves of wild strawberry, with yields ranging from 4.5% to 7.5%. Hydrolysate analysis revealed the presence of galactose, fructose, glucose, arabinose, xylose, and rhamnose, as well as glucuronic and galacturonic acids. The predominant monomeric components of the polysaccharide fraction were identified as glucose (6.2–10.0%), fructose (5.9–9.5%), glucuronic acid (11.3–24.6%), and galacturonic acid (5.7–12.6%). In addition to bound forms, free monosaccharides such as fructose, glucose, sucrose, and trehalose were also detected. Furthermore, the samples contained pectins and hemicelluloses, contributing to the structural and functional matrix of the plant tissues. Amino acid profiling revealed the presence of several proteinogenic amino acids, including aspartic acid, threonine, serine, glutamic acid, glycine, alanine, valine, methionine, leucine, tyrosine, lysine, phenylalanine, histidine, and arginine. The total content of free amino acids was quantified at 14 mg%, while the bound fraction reached 90 mg% [7].

Moreover, two novel compounds: 5-(4-hydroxy-3-methoxyphenethyl)-7-methoxy-2H-chromen-3-ol and 5-(4-hydroxy-3-methoxyphenethyl)-4,7-dimethoxy-2H-chromen-3-ol, were isolated from the ethyl acetate fraction of a methanolic extract derived from the aerial parts of *F. vesca* collected in Pakistan. Their structures were elucidated using a combination of infrared (IR), UV, and nuclear magnetic resonance (^1^H-NMR, ^13^C-NMR), supported by two-dimensional (2D) NMR techniques, including HMBC, HMQC, and COSY [81].

## 7. Biological Activity of *Fragaria vesca* and *Fragaria viridis* and Prospects for Their Application

The fruits and vegetative organs of *F. vesca* and *F. viridis* exhibit a broad spectrum of pharmacological activities. This is primarily due to their high content of bioactive compounds. These include polyphenols, triterpenoids, flavonoids, anthocyanins, organic acids, ascorbic acid, and aromatic compounds. Both species demonstrate significant antioxidant properties, which can be attributed to their elevated levels of ellagitannins, ellagic acid, gallic acid, anthocyanins, and ascorbic acid. These compounds effectively neutralize free radicals and protect cells from oxidative stress. Flavonoids and phenolic acids in strawberries possess the ability to inhibit inflammatory mediators. Additionally, the antimicrobial activity of extracts has been established, particularly against Gram-positive bacteria and certain fungi. This suggests their potential application in treating oral and skin inflammations. The antioxidants present in the berries contribute to protecting the liver from toxic damage. They also promote the health of the gastric mucosa. As a result, they help reduce the risk of ulcer formation. These beneficial effects are largely attributed to ellagic acid and flavonoids. Studies indicate that strawberry extracts can reduce blood glucose levels. They also improve insulin sensitivity and lower lipid levels. These effects are primarily attributed to polyphenols, especially quercetin and ellagitannins. Ellagic acid and its derivatives have been shown to inhibit tumor cell proliferation, induce apoptosis, and suppress angiogenesis. These effects have been extensively studied in models of breast, prostate, and colorectal cancers. Phenolic compounds play a crucial role in protecting neurons from oxidative damage. Because of this, both species are promising candidates for preventing age-related neurodegenerative diseases, such as Alzheimer’s disease.

An overview of the main biological effects of *F. vesca* and *F. viridis* is provided in Table 3.

Thus, *F. vesca* and *F. viridis* represent promising candidates for further investigation. Their high content of biologically active compounds, along with pronounced antioxidant, anti-inflammatory, and antimicrobial properties of the extracts, support their potential therapeutic relevance. These findings provide a strong rationale for continued in-depth study. Moreover, it was established that the LD_50_ of the ethanolic extract derived from *F. vesca* leaves exceeds 2000 mg/kg. According to established toxicological classifications, this places the extract in Class V, indicating it is practically non-toxic.

Currently, the development of the aforementioned formulations is underway, primarily based on *F. vesca.* For instance, a gel containing a 2% *F. vesca* leaf extract has been formulated as a promising cosmetic product with antioxidant and potential skin-brightening properties. It is suitable for safe and effective topical application. The gel demonstrated high antioxidant activity, comparable to that of ascorbic acid. Specifically, it reduced reactive oxygen species (ROS) levels by 86.2% under UV irradiation and by 61.1% following exposure to H_2_O_2_. In vitro studies using HaCaT keratinocyte cell lines confirmed the absence of cytotoxic effects. Furthermore, dermatological assessments conducted on human volunteers revealed good skin tolerance and no signs of irritation. The gel also maintained physicochemical stability under various storage conditions, including fluctuations in temperature and light exposure [23]. In addition, *F. vesca* fruit juice has been employed in the formulation of a cosmetic face powder. The study results indicated that the product was homogeneous, stable, non-irritating, and safe for use on the skin. The pH remained within the physiologically acceptable range of 4.5 to 6.5. The formulation was also well-received by study participants, indicating high user acceptability [103].

Turkish researchers emphasized the potential of *F. vesca* as a fruit capable of contributing substantially to regional economic development, particularly through commercial and gastronomic tourism. The incorporation of *F. vesca*-based dishes and beverages into local food service offerings is expected to generate significant added value for the region’s culinary sector [104]. In addition, commercially available herbal toothpaste containing *F. vesca* extract demonstrated notable efficacy in reducing bacterial accumulation on dental surfaces. It also promotes overall oral health [105].

Based on the chemical composition and demonstrated biological activity of *F. vesca* and *F. viridis*, a wide range of therapeutic and preventive formulations may be developed. These include antioxidant complexes (e.g., capsules, tablets) intended for the prevention of disorders associated with oxidative stress, such as cardiovascular diseases and neurodegenerative conditions. In addition, anti-inflammatory phytopharmaceuticals, such as syrups, tinctures, and drops, may be formulated for the treatment of chronic inflammatory disorders. Extract-based hepatoprotective agents designed to support liver function also represent a promising direction. Furthermore, anti-aging creams and serums exhibiting antioxidant activity could be developed for cosmetic purposes, including the reduction of wrinkles and dull skin tone. Other potential applications include the development of parapharmaceuticals and dietary supplements aimed at enhancing general health and well-being.

In conclusion, *F. vesca* and *F. viridis* should be regarded not only as edible plants but also as potential therapeutic agents. Although both species exhibit similar biological effects, *F. vesca* demonstrates comparatively stronger antioxidant and antimicrobial activities. In contrast, *F. viridis* may offer greater efficacy in managing diabetes and metabolic syndrome, likely due to differences in specific polyphenol and organic acid content. The results of recent studies confirm that both *F. vesca* and *F. viridis* are rich sources of biologically active compounds, including phenolic constituents, flavonoids, and vitamins. The observed antioxidant, anti-inflammatory, and antimicrobial properties of their extracts underscore their potential for application in the pharmaceutical, cosmetic, and functional food industries. Particularly promising is the development of natural phytopreparations aimed at preventing inflammatory conditions and enhancing the body’s antioxidant defense systems. Future research will focus on standardizing extracts, elucidating mechanisms of action, and designing innovative therapeutic and cosmetic products based on these plant species.

## Figures and Tables

**Table 1:** Chemical composition of essential oils of *Fragaria vesca* and *Fragaria viridis*.

Species	Location	Number of Identified Compounds	Main Compounds	Reference
*F. vesca*	Helsinki	99	c-decalacatone, c-dodecalactone, c-octalactone, d-decalactone, a-farnesene, a-pinene, α-terpineol, linalool, nerol, and myrtenol, methyl butanoate, 1-methylbutyl butanoate, hexyl butanoate, hexyl acetate, (Z)-3-hexenyl acetate, mesifurane, furaneol, € 2-pentenal, 1-penten-3-one	[32]
*F. viridis* (leaves and fruits)	Akmola region, Kazakhstan	39	β-linalool (0.8–8.9%), n-nonanal (0.5–8.6%), tetradecanal (2.1–5.9%), nerolidol (2.1–4.8%), unidentified component (1.9–6.6%), α-bisabolol (0.8–6.7%), phytol (18.4–47.4%), unidentified component (0.9–8.2%)	[33]
*F. viridis* (fruits)	Akmola region, Kazakhstan	34	m/p-xylene (2.4–14.0%), isoledene (4.7–8.5%), methyleugenol (3.3–8.4%), α-cedrene (2.5–3.9%), unidentified component (3.4–9.1%), α-muurolene (6.8–11.3%), nerolidol (1.1–4.8%), α-cedrol (1.7–8.0%), α-bisabolol (2.3–5.0%)	[33]
*F. vesca* (“Rugia” and “Baron von Solemacher” cv.) (leaves)	Poland	58	cumene (4.9–6.8%), linalool (13.4–14.1%), nonanal (18.7–20.1%), myrtenol (14.1–14.2%), citronellol (7.6–8.8%), geraniol (6.0–6.7%)	[34]
*F.viridis* (leaves)	East Kazakhstan	-	nonanal (2.5%), linalool (2.6%), dodecanoic (lauric) acid (3.4%), geranyl linalool (3.6%), phytol (17.6%), tetradecanoic (myristic) acid (4.8%), hexadecenoicoic (palmitic) acid (30.7%)	[35]
*F. vesca* (cultivar Hawaii 4)	China	141	ethyl butanoate (1.2%), butyl acetate (1.883%), hexyl acetate (1.2%), octyl acetate (2.874%), 2-nonyl acetate (1.243), decyl acetate (1.536), 2-heptanone (12.413), 2-nonanone (8.935)	[23]
*F. vesca* (cultivar Reugen)	China	139	butyl acetate (1.895%), hexyl acetate (1.771%), octyl acetate (5.657%), decyl acetate (1.536%), 2-heptanone (32.821%), 2-nonanone (16.439%)	[36]
*F. vesca* (cultivar Yellow Wonder)	China	165	ethyl butanoate (1.49%), butyl acetate (1.126%), hexyl acetate (1.255%), octyl acetate (3.156%), 2-heptanone (35.886%), 2-nonanone (12.81%)	[36]
*F. vesca*, cultivarUC4	Japan	57 identified esters	ethyl acetate (26.27%)	[37]
*F. vesca*, cultivar UC5	Japan	57 identified esters	octyl acetate (80.34%)	[37]
*F. vesca*, cultivar Jinchuan	Japan	57 identified esters	ethyl acetate (17.62%), 1-methyl tridecyl acetate (20.26%), ethyl octanoate (14.53%)	[37]
*F. vesca*, cultivar Maoxian	Japan	57 identified esters	myrtenyl acetate (5.49%)	[37]
*F. vesca*, cultivar Northeast Wild	Japan	57 identified esters	octyl acetate (12.98%), 1-methyl tridecyl acetate (39.47%), ethyl hexanoate (14.75%)	[37]
*F. vesca*, cultivar Fifteen Kuang	Japan	57 identified esters	ethyl acetate (45.77%)	[37]
*F. vesca*, cultivar Mean	Japan	57 identified esters	ethyl acetate (14.94%), octyl acetate (18.79%), 1-methyl tridecyl acetate (10.84%)	[37]
*F. vesca*	Finland	87	2,5-dimethyl-4-methoxy-3(2H)-furanone	[38]
*F. vesca*, cultivar Regina delle Valli	Italy	131	methyl anthranilate	[39]
*F. vesca*	Sweden	24	α-muurolene (18.5%), benzaldehyde (14.5%)	[40]
*F. vesca,* cultivar ‘Yellow Wonder	Korea	53	ethyl butanoate, 1-hexanol, hexyl acetate	[38]
*F. vesca,* cultivar Baron Solemacher’	Korea	44	ethyl butanoate, hexyl acetate	[41]
*F. vesca*	Finland	58	2, 5-dimethyl-4-methoxy-3(2fl)-furanone	[42]

**Table 2:** Combinations of anthocyanidins and functional groups determining color expression in *Fragaria* species.

Name	Position of Attachment to a Molecule							Color
	3	5	6	7	3′	4′	5′	
Cyanidin	OH	OH	H	OH	OH	OH	H	Red-orange
Delphinidin	OH	OH	H	OH	OH	OH	OH	Red-blue
Malvinidin	OH	OH	H	OH	OCH_3_	OH	OCH_3_	Red-blue
Pelargonidin	OH	OH	H	OH	H	OH	H	Orange
Peonidin	OH	OH	H	OH	OCH_3_	OH	H	Red-orange
Petunidin	OH	OH	H	OH	OCH_3_	OH	OH	Red-blue

**Table 3:** Biological activity of *Fragaria vesca* and *Fragaria viridis*.

Biological Activity	Properties of the Sample	Source
Antioxidant and antiradical	*F. vesca* from Serbia showed strong antioxidant activity (DPPH, FRAP)—87.12 mg AA·g^−1^ DW	[82]
	*F. vesca* from Slovakia antioxidant activity (phosphomolybdenum method)—679.56 ± 3.06 mg TE/g)	[80]
	Fruit extract of *F. vesca* in DPPH assay—53.92–87.17%, compared to *F. × ananassa* 27.21–52.58%	[83]
	*F. viridis* extracts (all ripening stages) showed strong antioxidant activity: ABTS—35.07–36.22, DPPH—27.53–29.18 µM TE·g^−1^ DW; *F. vesca* showed lower activity: ABTS—19.73, DPPH—15.21 µM TE·g^−1^ DW	[55]
	Wild F. *vesca* infusion: highest phenolic content 253.42 mg/g DW; strong antioxidant activity—DPPH, reducing capacity, lipid peroxidation EC_50_ = 50.56, 44.78, 4.76 µg/mL	[84]
	*F. vesca* extract: in SW480 cells—G2 phase arrest; in E705 cells—apoptosis via ROS increase.	[85]
	*F. vesca* extract from Bulgaria: high antioxidant activity in ABTS assay—3.74 ± 0.06 mM	[86]
	*F. vesca* extract showed highest antioxidant activity in vivo and in vitro, linked to DPPH scavenging, tannins, and antioxidant gene regulation	[87]
	White *F. vesca* fruits had higher antioxidant activity than red; all extracts reduced oxidative damage via direct antioxidants and enzyme action	[88]
	*F. vesca* showed strong antioxidant activity in OxHLIA, indicating cell protection from oxidative stress	[89]
	The leaf extracts of *F. vesca* tested demonstrated considerable free radical scavenging ability at higher concentrations	[90]
	F. *vesca* from Iran showed strong antioxidant activity, highly correlated with phenolics (r = +0.99) and anthocyanins (r = +0.93); biochemical content linked to climate factors	[91]
Anti-inflammatory	Ethanolic *F. vesca* extract (500 mg/kg) improved inflammatory bowel disease in vivo, likely due to antioxidant and anti-inflammatory effects	[92]
	*F. vesca* leaf extract significantly reduced Freund’s adjuvant-induced edema (1.3–5×) and indomethacin-enhanced edema (1.6–3.8×); also decreased hyperemia and granulomatous tissue in inflammation models	[7]
	The leaf extract of *F. vesca* inhibited cyclooxygenase activity in in vitro experiments	[93]
	*F. vesca* extract (80–160 mg/mL) reduced nitrite production, inhibited proteasome activity causing ubiquitinated protein buildup, and induced autophagy (LC3-I to LC3-II conversion)	[67]
Antimicrobial	*F. vesca* extract (0.08 g/L) demonstrated strong synergy with colistin (4 mg/L) in inhibiting a colistin-resistant phenotype of *Pseudomonas aeruginosa*	[22]
	The alcoholic extract of *F. vesca* leaves exhibited notable antibacterial activity against *P. aeruginosa* producing the metallo-β-lactamase VIM-2	[94]
	*F. viridis* leaf infusions reduced *Escherichia coli* growth 2–10 fold in all tested samples	[95]
	*F. vesca* crude extract and ellagitannin fraction showed antimicrobial activity against *Helicobacter pylori*, with the fraction inhibiting 67% at 5 mg/mL and crude extract 58% at 12.5 mg/mL	[96]
Analgesic	*F. vesca* leaf extract showed significant analgesic effects, increasing pain response latency 1.9–2.3× in healthy and inflamed animals, and reducing pain in writhing and hot plate tests across inflammation models	[96]
Antifungal	Aqueous *F. vesca* extracts showed strong antifungal activity with minimal inhibitory concentrations of 25–50 mg/mL against yeast-like fungi	[97]
Diuretic	*F. vesca* leaf infusion (150 mg/kg) showed diuretic effects, increasing urine output by 2.9 mL at 24 h and 1.7 mL over the following 19 h	[98]
	*F. vesca* leaf phenolics (80% ethanol extract) increased diuresis twofold at 50 mg/kg and threefold at 100 mg/kg within 4 h, peaking in the first hour	[7]
Antidiabetic	*F. vesca* extract inhibited α-amylase and α-glucosidase by 96% and 97% at 5 mg/mL	[99]
Antitumor	Ellagitannin-enriched fraction from *F. vesca* leaves showed stronger cytotoxicity than crude extract in HepG2 cells, causing G2/M arrest, autophagy inhibition, ubiquitin-proteasome system impairment, and altered metabolic proteins, indicating therapeutic potential for liver cancer	[78]
Antimelanogenic effect	*F. vesca* leaf extract inhibited the target enzyme with IC_50_ = 238.10 ± 15.51 µg/mL, comparable to arbutin (IC_50_ = 193.84 ± 14.15 µg/mL)	[23]
Cytotoxicity	*F. vesca* leaf extract (2 mg/mL) significantly reduced HaCaT keratinocyte viability, indicating cytotoxicity	[23]
	*F. vesca* leaf extract affected keratinocyte and fibroblast metabolism, proliferation, and migration in vitro	[90]
Anticoagulant activity	*F. vesca* extracts and fractions showed anticoagulant activity by inhibiting the intrinsic pathway in activated partial thromboplastin time assay; purified fractions had the strongest effects	[29]
Hypolipidemic	Ethanolic (250–500 mg/kg) and aqueous (500 mg/dL) *F. vesca* fruit extracts showed significant hypolipidemic activity; 500 mg/kg ethanolic extract matched atorvastatin in vivo	[100]
Antitubercular	*F. vesca* extract (10%) strongly inhibited growth of *Mycobacterium tuberculosis* complex strains	[101]
Effects on the cardiovascular system	*F. vesca* leaf aqueous extract caused dose-dependent, endothelium-dependent vasodilation via NO stimulation without affecting heart rate or contractility	[63]
	The hydroalcoholic extract of *F. vesca* did not significantly alter basal vascular tone (E_max_ = 0.62 ± 0.48 mN, *n* = 3); however, the leaf extract substantially potentiated the contractile response to norepinephrine	[102]
Antidepressant effect	Two benzyl derivatives from *F. vesca* var. nubicola Lindl. ex Hook.f. showed significant antidepressant-like effects in tail suspension and forced swim tests	[81]

## Data Availability

No new data were created or analyzed in this study.
